# *FST* Polymorphisms Associate with Musculoskeletal Traits and Modulate Exercise Response Differentially by Sex and Modality in Northern Han Chinese Adults

**DOI:** 10.3390/genes16070810

**Published:** 2025-07-10

**Authors:** Wei Cao, Zhuangzhuang Gu, Ronghua Fu, Yiru Chen, Yong He, Rui Yang, Xiaolin Yang, Zihong He

**Affiliations:** 1 Comprehensive Experimental Center, China Institute of Sport Science, Beijing 100061, China; cw0616@126.com (W.C.); ronghuafu1998@163.com (R.F.); tong_lucky@163.com (Y.C.); he18263825095@163.com (Y.H.); candyyeung0825@163.com (R.Y.); 2 School of Kinesiology, Shanghai University of Sport, Shanghai 200438, China; 3 Institute of Physical Education, Henan Normal University, Xinxiang 453007, China; 2023292@htu.edu.cn; 4 China Institute of Sport and Health Science, Beijing Sport University, Beijing 100091, China; yangxiaolin@bsu.edu.cn

**Keywords:** follistatin gene, single nucleotide polymorphisms, exercise intervention, body composition, bone mineral content

## Abstract

**Background/Objectives**: To investigate associations between Follistatin (*FST*) gene polymorphisms (SNPs) and baseline musculoskeletal traits, and their interactions with 16-week exercise interventions. **Methods**: A cohort of 470 untrained Northern Han Chinese adults (208 males, 262 females), sourced from the “Research on Key Technologies for an Exercise and Fitness Expert Guidance System” project, was analyzed. These participants were previously randomly assigned to one of four exercise groups (Hill, Running, Cycling, Combined) or a non-exercising Control group, and completed their respective 16-week protocols. Body composition, bone mineral content (BMC), bone mineral density (BMD), and serum follistatin levels were all assessed pre- and post-intervention. Dual-energy X-ray absorptiometry (DXA) was utilized for the body composition, BMC, and BMD measurements. *FST* SNPs (rs3797296, rs3797297) were genotyped using matrix assisted laser desorption/ionization time-of-flight mass spectrometer (MALDI-TOF MS) or microarrays. To elucidate the biological mechanisms, we performed in silico functional analyses for rs3797296 and rs3797297. **Results**: Baseline: In females only, the rs3797297 T allele was associated with higher muscle mass (β = 1.159, 95% confidence interval (CI): 0.202–2.116, *P*__adj_ = 0.034) and BMC (β = 0.127, 95% CI: 0.039–0.215, *P__adj_* = 0.009), with the BMC effect significantly mediated by muscle mass. Exercise Response: Interventions improved body composition, particularly in females. Gene-Exercise Interaction: A significant interaction occurred exclusively in women undertaking hill climbing: the rs3797296 G allele was associated with attenuated muscle mass gains (β = −1.126 kg, 95% CI: −1.767 to −0.485, *P*__adj_ = 0.034). Baseline follistatin correlated with body composition (stronger in males) and increased post-exercise (primarily in males, Hill/Running groups) but did not mediate SNP effects on exercise adaptation. Functional annotation revealed that rs3797297 is a likely causal variant, acting as a skeletal muscle eQTL for the mitochondrial gene *NDUFS4*, suggesting a mechanism involving muscle bioenergetics. **Conclusions**: Findings indicate that *FST* polymorphisms associate with musculoskeletal traits in Northern Han Chinese. Mechanistic insights from functional annotation reveal potential pathways for these associations, highlighting the potential utility of these genetic markers for optimizing training program design.

## 1. Introduction

Abnormal body composition—including excess fat mass, reduced muscle mass, and low bone mass—is associated with obesity, osteoporosis, and various chronic diseases. Research indicates that fat mass and muscle mass exert contrasting effects on health outcomes: higher fat mass correlates positively with all-cause mortality [1,2], whereas greater muscle mass often provides protective benefits [3,4]. Thus, maintaining appropriate levels of body fat and skeletal muscle is crucial for reducing mortality risk. Concurrently, reduced bone mineral density (BMD) and bone mineral content (BMC) significantly increase the risk of osteoporosis [5,6], thereby diminishing quality of life. Given these associations, precise evaluation and effective interventions to improve body composition are essential public health priorities.

Exercise is widely regarded as an effective strategy to improve body composition, reducing fat mass, increasing muscle mass, and improving bone mass [7,8]. However, inter-individual variability in response to exercise is pronounced [9,10], with evidence suggesting that genetic factors play a crucial role [11]. These genetic effects are likely mediated through complex interactions with environmental factors, including the specific type of exercise performed [12,13]. Different exercise modalities, such as hill climbing, running and cycling, or combined training, elicit distinct physiological demands and adaptive outcomes that uniquely influence body composition [14,15]. For instance, hill climbing emphasizes eccentric loading, which is beneficial for muscle hypertrophy [16]. Running, a high-impact and weight-bearing activity, generates repetitive ground reaction forces crucial for stimulating bone mineral density [17]. In contrast, cycling is a low-impact exercise that provides less stimulation for bone density and thus may not confer the same osteogenic benefits [18]. While these individual modalities offer distinct physiological adaptations, combined training—often incorporating both endurance and resistance elements—can provide a broader range of stimuli to the musculoskeletal system, potentially yielding more holistic benefits than any single discipline [19]. Therefore, investigating how genetic predispositions modulate responses to these diverse training stimuli is essential for developing more effective, personalized exercise strategies.

Follistatin (*FST*) is an autocrine glycoprotein extensively involved in regulating muscle, adipose, and bone metabolism [20,21]. Acting as an endogenous antagonist, *FST* binds members of the transforming growth factor-beta (TGF-β) superfamily—such as myostatin, activin A, and growth differentiation factor 11 (GDF11)—to prevent their interaction with the activin receptor type IIB (ActRIIB), thereby promoting muscle fiber hypertrophy and protein synthesis [22]. Additionally, *FST* indirectly enhances BMP-mediated Smad1/5/8 signaling, facilitating anabolic processes in skeletal muscle [23]. BMPs also regulate adipocyte differentiation and thermogenesis, promoting fat browning [24], suggesting that *FST*’s inhibition of TGF-β signaling may foster adipocyte differentiation and browning, thereby modulating overall energy metabolism [20,25]. Furthermore, *FST*’s inhibition of activin and myostatin signaling in osteocytes contributes to maintaining bone mass [26]. This complex regulatory role implies that genetic variations within the *FST* gene could influence muscle mass, adipose tissue metabolism, and bone metabolism, leading to distinct physiological responses to various exercise interventions.

Previous research has begun to explore the role of *FST* gene variants, though comprehensive findings across diverse populations and exercise types remain scarce. Studies in European cohorts, for example, have linked certain *FST* gene polymorphisms to variations in lean mass [27]. Specifically, the T allele of *FST* rs3797297 has been identified as a lean mass–increasing allele in the UK Biobank cohort (*p* = 9.9 × 10^−21^; n = 450,243) [27], and as a predisposing allele for baseline isometric knee extensor strength and detraining-related strength changes in older Flemish Caucasians [28]. These findings highlight the potential impact of *FST* gene variants on musculoskeletal traits and exercise adaptability. However, while initial studies have explored *FST* gene variants’ associations with muscle size and strength, their broader effects on comprehensive body composition indicators—such as muscle mass, fat mass, BMD, and BMC—across various exercise modalities require more thorough investigation.

Therefore, this study aimed to (1) investigate associations of *FST* single nucleotide polymorphisms (SNPs) (rs3797296, rs3797297) with baseline body composition and bone mineral parameters in Northern Han Chinese adults; (2) examine the interactions between these SNPs and the effects of different 16-week exercise interventions (Hill, Running, Cycling, Combined) on these parameters; and (3) explore the potential mediating role of serum follistatin in these relationships. By elucidating how *FST* genetics influence baseline traits and modulate responses to various exercise types, this research seeks to provide insights into gene-exercise interactions and contribute foundational knowledge towards developing more personalized and effective exercise strategies for optimizing body composition and musculoskeletal health.

## 2. Materials and Methods

### 2.1. Participants and Study Design

Participants in this study were included from an established sample and DNA repository, developed under the project “Research on Key Technologies for an Exercise and Fitness Expert Guidance System (2012BAK23B01)” [29]. Inclusion criteria were (1) age 18–69 years, (2) Han Chinese ancestry without close kinship with other participants, (3) free of cardiovascular disease, diabetes, or other acute or chronic diseases, (4) no regular physical training in the past year (less than two exercise sessions per week, each <20 min, or total weekly exercise <75 min), and (5) no smoking or heavy alcohol use. Exclusion criteria included: (6) pregnancy or lactation; (7) any musculoskeletal injury or condition preventing participation in exercise; (8) use of medications known to affect muscle, bone, or metabolism; (9) participation in other concurrent research studies; or (10) any medical condition contraindicating exercise. A total of 470 participants (208 males, 262 females) who completed the 16-week interventions with complete genotype and body composition data were included in analyses of *FST* SNPs, baseline body composition, and exercise response. Among these, 234 participants (114 males, 120 females) had complete serum follistatin data before and after intervention, providing baseline levels and post-training changes. Finally, mediation analyses were conducted in this subset to examine potential mediator roles of follistatin in the relationship between *FST* SNPs and changes in body composition or bone mineral parameters after 16 weeks.

### 2.2. Ethics Statement

This study protocol was reviewed and approved by the Ethics Committee of the China Institute of Sports Science (CISS-2013-13) on 9 April 2013. All procedures performed were in accordance with the ethical standards of the Declaration of Helsinki. Prior to participation, all participants were fully informed about the study’s procedures and potential risks, and subsequently provided their written informed consent.

### 2.3. Exercise Intervention Protocols

**Figure 1:**
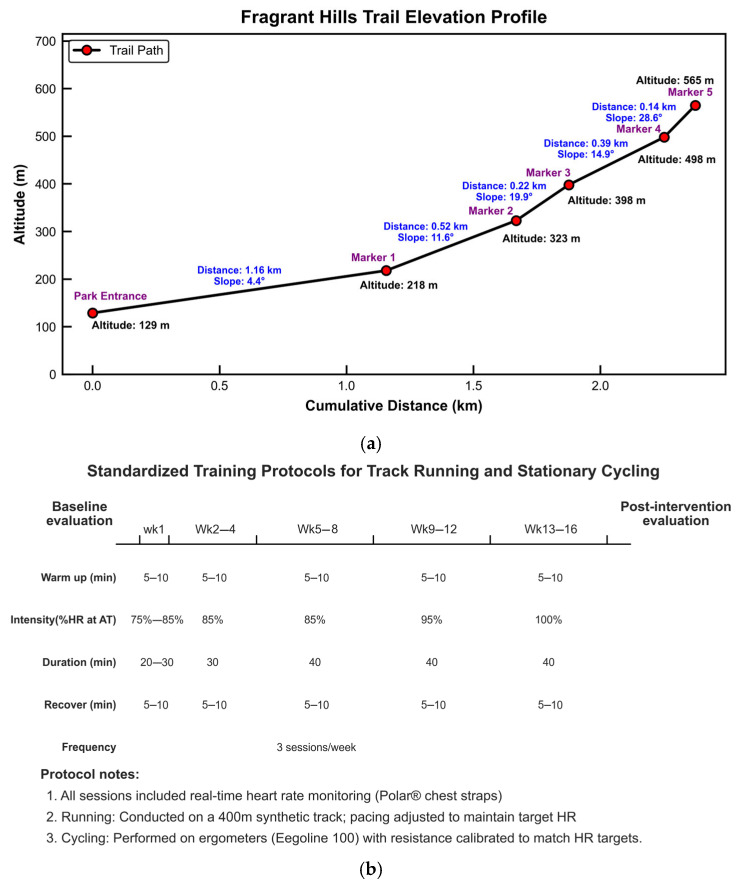

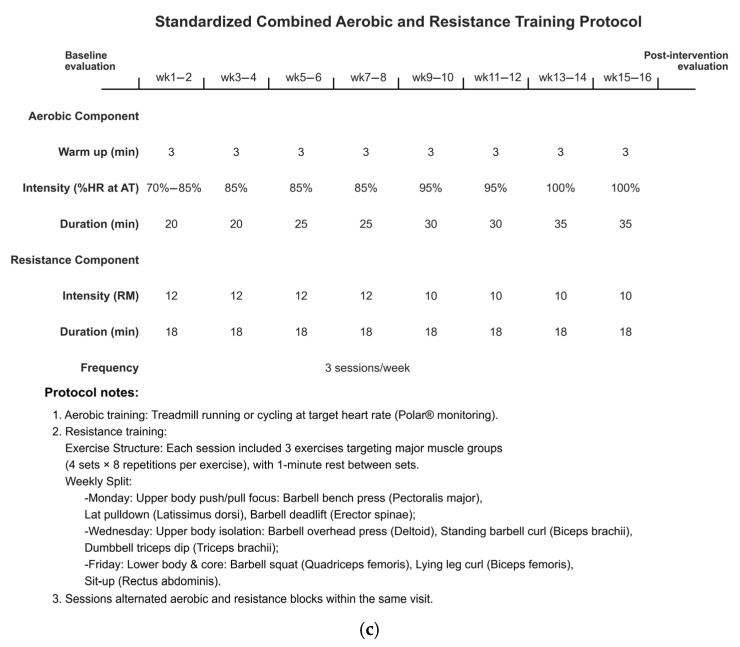
Overview of the exercise intervention programs. (**a**) Hill climbing route and elevation profile. (**b**) Endurance exercise training program (Running or Cycling). (**c**) Combined endurance and strength training program.

Participants were randomized into five groups for a 16-week intervention (Figure 1): (1) Hill Climbing group (Hill; n = 64): Completed weekly outdoor hill climbs (Fragrance Hill, Beijing; altitude 565 m, ~2.43 km) for 16 weeks, ascending within 2 h per session. (2) Running group (Running; n = 150): Completed three weekly outdoor track running sessions on a standardized 400 m athletic field for 16 weeks (protocol details in Figure 1b). (3) Cycling group (Cycling; n = 133): Completed three stationary cycling sessions weekly for 16 weeks (protocol details in Figure 1b). (4) Combined Training group (Combined; n = 49): Engaged in three weekly sessions integrating aerobic exercise and resistance training for 16 weeks (Figure 1c). (5) Control group (Control; n = 74): Maintained habitual physical activity without structured exercise. Compliance and adverse events were monitored throughout the intervention.

The exercise modalities were chosen to reflect common real-world practices. The differing session frequencies and durations (e.g., Hill: 1 × 120 min/week vs. Running/Cycling: 3 × 40 min/week) were standardized within each group by design to enhance ecological validity and adherence (Hill) or align with public health guidelines for cardiorespiratory fitness (Running/Cycling). While total exercise dose (volume and intensity) varied across intervention groups due to these distinct protocols, each modality’s intervention was rigorously controlled and standardized throughout the 16 weeks, focusing on adherence to its specific intensity targets and duration.

### 2.4. Anaerobic Threshold Testing

All participants underwent a graded cycle ergometer test (Eegoline100, Ergoline Academy, Germany) coupled with a cardiopulmonary measurement system (MataMax3B, Cortex, Germany) to determine anaerobic threshold (AT). Males started at 25 W, females at 20 W; the load increased by 25 W or 20 W every 2 min, pedaling at 60 rpm until volitional exhaustion. The V-slope method [30] was used to identify AT via the breakpoint in the VCO_2_–VO_2_ relationship. AT (in L·min^−1^) was the primary measure for setting and monitoring exercise intensity.

### 2.5. Body Composition Assessment

Dual energy X-ray absorptiometry (DXA) (GE LUNAR DPX system, Madison, WI, USA) was used to measure total body fat mass (kg), muscle mass (kg), bone mineral density (BMD, g/cm^3^), and bone mineral content (BMC, kg) before and after the 16-week intervention. Participants removed metal accessories and wore light clothing, lying supine with arms alongside the body while the legs were stabilized to ensure accurate measurements. They were advised to refrain from intense exercise or heavy meals before DXA scans.

### 2.6. DNA Extraction and Genotyping

For genetic variant selection, tag single nucleotide polymorphisms (tagSNPs) within the *FST* gene were identified using the 1000 Genomes Project (Phase 3, GRCh37) database [31] as a reference resource for human genetic variation. Specifically, we retrieved chromosome 5 data for the *FST* gene region (Chromosome 5: 52,776,239–52,782,964) from the Han Chinese in Beijing (CHB) population (n = 103). To ensure high-quality data for tagSNP selection, initial filtering was performed using PLINK software (v1.90) [32] based on minor allele frequency (>0.01), SNP call rate (>0.95), and Hardy–Weinberg equilibrium (HWE) (*p*-value > 1 × 10^−6^). This process yielded a set of 12 SNPs, which were then analyzed using Haploview software (v4.2) [33]. TagSNPs were identified with a linkage disequilibrium (LD) threshold of r^2^ ≥ 0.8 to effectively capture the genetic variation in the *FST* gene.

Following tagSNP selection, further refinement was performed based on their merging status in the National Center for Biotechnology Information (NCBI) database and their reported functional relevance or prior association with musculoskeletal traits in the existing literature [28]. Ultimately, three SNPs (rs12152850, rs3797296, rs3797297) were selected for genotyping in our study cohort.

For our study cohort’s genotyping, Peripheral venous blood (10 mL) was collected from each participant, and DNA was extracted using the TIANGEN Genomic DNA Extraction Kit (DP348, Beijing, China). Genotyping was performed using matrix-assisted laser desorption/ionization time-of-flight mass spectrometry (MALDI-TOF MS, Bruker Daltonics GmbH, Bremen, Germany), with a success rate >95%.

### 2.7. Serum Follistatin Measurement

Blood samples (10 mL) were collected by venipuncture in the morning following an overnight fast (no strenuous exercise on the preceding day). After immediate centrifugation (3000 rpm, 10 min), serum was separated and stored at −80 °C. Serum follistatin levels were measured using an ELISA kit (DFN00, R&D Systems, Minneapolis, MN, USA), with intra-assay and inter-assay coefficients of variation ≤ 2.7% and ≤ 9.2%, respectively. A standard curve was generated using duplicate parallel wells for improved precision.

### 2.8. In Silico Functional Annotation

To investigate the potential biological functions of the *FST* variants rs3797296 and rs3797297, a series of bioinformatic analyses were performed. Basic variant information, including genomic location and functional consequence, was retrieved from the NCBI dbSNP database [34]. Linkage disequilibrium (LD) between the two SNPs was assessed using the LDpair module of LDlink v4.2 [35], based on data from the 1000 Genomes Project Phase 3 for the CHB population.

We queried several large-scale public databases to evaluate previously reported genetic associations. These included the NHGRI-EBI GWAS Catalog [36], the ClinVar database [37] for clinical significance, and the Open Targets Genetics portal [38] for comprehensive phenome-wide association studies (PheWAS) summary statistics from sources such as the UK Biobank and FinnGen.

To assess the regulatory potential of these SNPs, we utilized the Genotype-Tissue Expression (GTEx) portal [39] to identify expression quantitative trait loci (eQTLs) and splicing quantitative trait loci (sQTLs) across all available tissues, with a particular focus on skeletal muscle. Finally, to explore epigenetic context, we used the HaploReg [40] and RegulomeDB [41] databases to annotate the variants for chromatin states, histone modifications, transcription factor binding sites (TFBS), and DNAse I hypersensitivity sites derived from the ENCODE and Roadmap Epigenomics projects.

### 2.9. Statistical Analysis

All statistical analyses were performed in Python 3.12. Continuous variables were tested for normality using the Shapiro–Wilk test. Normally distributed data are presented as mean ± standard deviation (SD), while skewed data are presented as median (interquartile range, IQR). Baseline characteristics were compared between sexes using independent t-tests for normally distributed variables or Mann–Whitney U-tests for non-normal distributions. Hardy–Weinberg equilibrium (HWE) was tested by chi-square analysis. Additionally, allele frequencies between our study cohort and the 1000 Genomes CHB population were compared using chi-square tests for homogeneity.

We assumed a dominant genetic model for each SNP and used generalized linear models (GLM) to examine associations between *FST* SNPs and baseline body composition/bone mineral parameters, adjusting for age and baseline BMI. For BMD and BMC, we further adjusted for muscle mass or fat mass to explore potential influences. Mediation analysis (bootstrap resampling 5000 times) was used to determine whether muscle or fat mass mediated SNP effects on BMD/BMC, adjusting for age and baseline BMI.

To assess within-group changes in body composition, bone mineral parameters, and follistatin levels from baseline to post-intervention, paired t-tests (for normally distributed data) or Wilcoxon signed-rank tests (for non-normally distributed data) were used. These analyses were conducted within each individual exercise group (Hill, Running, Cycling, Combined), the control group, and also for the pooled exercise group (combining data from all four exercise modalities). To assess differences between the pooled exercise group and the control group, as well as among the four distinct exercise modalities (Hill, Running, Cycling, Combined) versus control, GLM were employed, adjusting for age and baseline BMI, ensuring independent comparisons for each modality. However, direct comparisons of efficacy between different exercise modalities should be interpreted cautiously due to the intentional variation in exercise dose parameters across groups, as described in the Exercise Intervention Protocols section. Our analyses focused primarily on comparing each exercise modality to the control group and on examining genotype-by-exercise modality interactions, rather than on making direct comparisons of effectiveness between different exercise types. Genotype-by-intervention interactions, assessing whether genetic polymorphisms moderated exercise responses, were examined using GLM. These models utilized the 16-week change values as the dependent variable, with main effects of SNPs, intervention group status, and their interaction terms included as predictors. For these interaction analyses, intervention group status was defined in two ways: (1) comparing the pooled exercise group versus the control group, and (2) comparing each exercise modality individually versus the control group.

Among the 234 participants with serum follistatin data, Pearson or Spearman correlations assessed the relationships between serum follistatin levels and baseline body composition and bone mineral indicators. Changes in follistatin levels before and after the 16-week intervention were compared using paired t-tests (for normally distributed data) or Wilcoxon signed-rank tests (for non-normally distributed data) for the pooled exercise group as well as for each exercise modality group (Hill, Running, Cycling, Combined).

Mediation analyses using GLM were conducted to explore whether serum follistatin mediated the relationship between *FST* SNPs and exercise responses after 16 weeks. Each mediation model set the change in exercise responses as the dependent variable, included *FST* SNPs and their interactions with intervention groups as independent variables, and used the change in follistatin levels as the mediator. Mediation effects were assessed by constructing regression models that included the mediation path and employed bootstrap resampling (5000 iterations) to calculate 95% confidence intervals, thereby evaluating the statistical significance and extent of the mediation effects.

Multiple comparisons were controlled using the Benjamini–Hochberg false discovery rate (FDR). The FDR level was set at 0.05. Adjusted *p* < 0.05 was considered statistically significant.

## 3. Results

### 3.1. Participant Characteristics

A total of 470 participants (208 males, 262 females) were included in the initial analysis (Chinese cohort). Baseline characteristics are summarized in Table 1, highlighting significant sex differences in body composition and bone mineral parameters (*p* < 0.05). Sex-stratified analyses were therefore conducted in subsequent steps.

Genotype information for *FST* SNPs is presented in Table 2. All three SNPs were found to be in Hardy–Weinberg equilibrium (*p* > 0.05 for all). In our cohort, the minor allele frequencies (MAFs) were 0.020 for rs12152850, 0.188 for rs3797296, and 0.124 for rs3797297. To confirm the representativeness of our study cohort, these MAFs were compared against reported frequencies for the Han Chinese in Beijing (CHB) population from the 1000 Genomes Project (1000G). The observed MAFs showed high consistency with the 1000G CHB population data (rs12152850: 0.015, rs3797296: 0.180, and rs3797297: 0.107). Chi-square tests for homogeneity further confirmed no statistically significant differences in allele frequencies between our cohort and the 1000G CHB population for any of the three SNPs (rs12152850: χ^2^ = 0.065, *p* = 0.799; rs3797296: χ^2^ = 0.037, *p* = 0.849; rs3797297: χ^2^ = 0.343, *p* = 0.558). This high degree of consistency suggests that our study cohort is representative of the broader CHB population for these specific genetic markers, thereby minimizing concerns regarding population stratification or sampling bias in our downstream analyses.

Due to the low frequency of the rs12152850 T allele (MAF = 0.020), subsequent analyses focused on the common variants rs3797296 and rs3797297 to maintain statistical power.

### 3.2. Associations of FST SNPs with Baseline Body Composition and Bone Mineral Parameters

#### 3.2.1. rs3797297 Association with Baseline Muscle Mass and BMC in Women

Associations of *FST* SNPs with baseline body composition and bone mineral parameters were analyzed using data from the full cohort (n = 470). Because initial analyses indicated no significant results in the total sample but notable sex differences (see Supplemental Table S1), subsequent GLM analyses were stratified by sex. In sex-stratified analyses, the *FST* rs3797297 T allele (GT/TT genotype) was significantly associated with higher baseline muscle mass and BMC in women (Table 3). Specifically, women with the GT/TT genotype had, on average, 1.159 kg greater muscle mass (95% CI: 0.202–2.116, *P*__adj_ = 0.034) and 0.127 kg greater BMC (95% CI: 0.039–0.215, *P*__adj_ = 0.009) compared to GG genotype carriers. A positive trend with BMD (*p* = 0.040) did not remain significant after adjustment. No significant associations were found for rs3797297 in men, or for rs3797296 with any baseline indicators in either sex (Supplemental Table S2).

#### 3.2.2. Mediation of Muscle Mass in rs3797297 Effect on BMC

Mediation analysis indicated that muscle mass significantly mediated the association between rs3797297 and BMC in women (n = 262) (Table 4). Supporting this, the significant positive association between rs3797297 and BMC in women was attenuated after adjusting for muscle mass (Supplemental Table S3).

### 3.3. Effects of the Exercise Intervention on Body Composition and Bone Mineral Parameters

**Figure 2:**
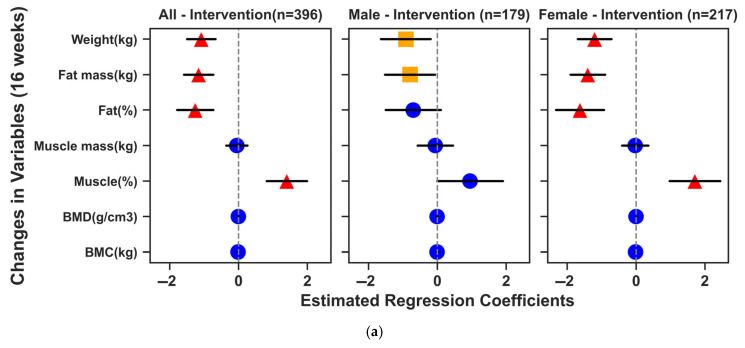

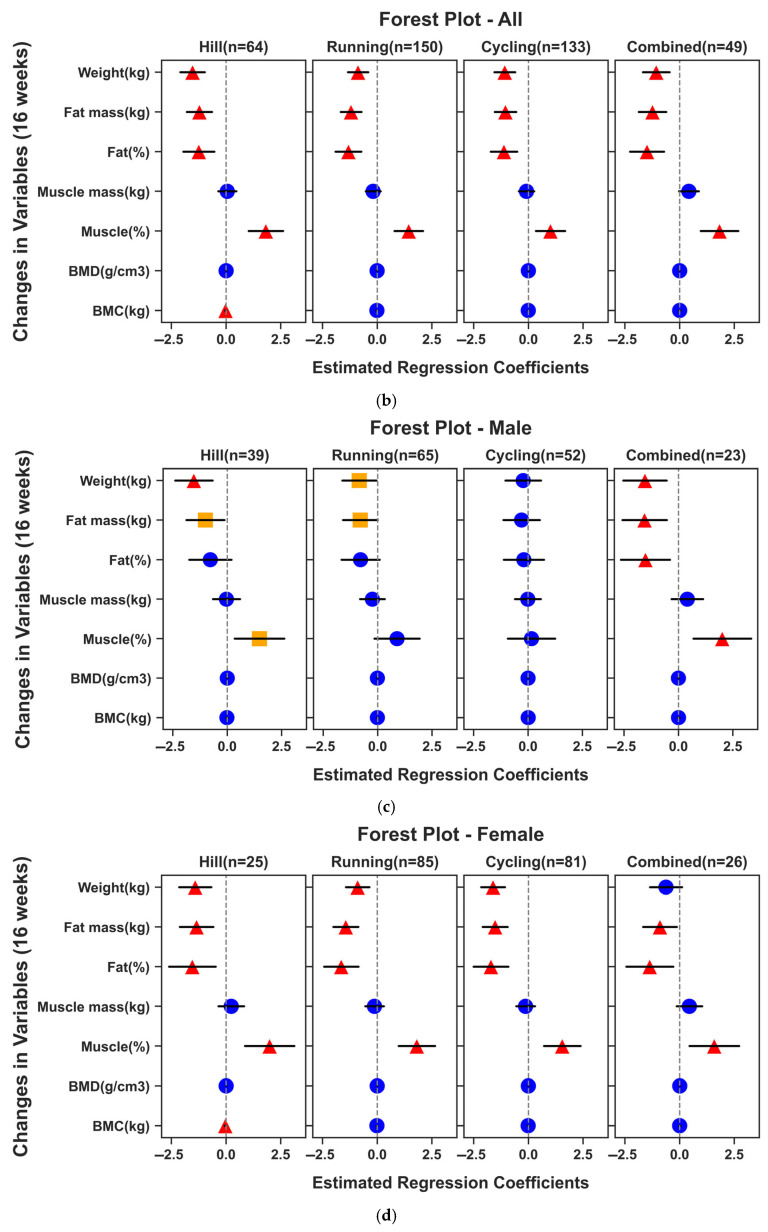
Effects of the 16-week exercise intervention on body composition and bone mineral parameters. (**a**) shows the results of GLM (adjusted for age, baseline BMI, and corresponding baseline values) analyzing the impact of a 16-week exercise intervention on changes in body composition and bone mineral parameters. b, c, and d depict how different exercise modalities (Hill, Running, Cycling, Combined) influenced changes in these parameters among all participants (**b**), men (**c**), and women (**d**), respectively, under the same covariate adjustments. Dots and error bars represent regression coefficient estimates and their 95% confidence intervals, color-coded to indicate statistical significance (blue circles, nonsignificant; orange squares, significant before adjustment; red triangles, significant after Benjamini–Hochberg correction).

The effects of the 16-week exercise intervention on body composition and bone mineral parameters were analyzed in the full cohort (n = 470). Overall, the 16-week exercise intervention yielded significant improvements in body composition (Figure 2). Compared with controls, the pooled exercise group showed reduced weight, fat mass, and body fat percentage, along with increased muscle percentage. Stratified by sex, these changes were generally more pronounced in females (consistent with within-group comparisons, see Supplemental Table S4). When comparing specific exercise modalities with the control group, all four interventions significantly reduced weight, fat mass, and fat percentage and increased muscle percentage in the overall sample (Figure 2b). Among males, the Combined and Hill programs showed significant benefits for fat reduction and increasing muscle percentage (Figure 2c). Among females, all four exercise modalities demonstrated notable improvements in body composition (Figure 2d). Importantly, only the Hill intervention significantly increased BMC in the overall and female samples, highlighting its potential benefit for bone mineral content alongside favorable body composition changes. The within-group changes for each exercise modality were consistent with these results (see Supplemental Table S5).

### 3.4. Interactions of FST SNPs with the 16-Week Exercise Responses

Interactions of *FST* SNPs with the 16-week exercise responses were assessed in the full cohort (n = 470). No significant interactions between rs3797296 or rs3797297 and the overall exercise intervention (pooled exercise groups vs. control) were observed for changes in body composition or bone mineral parameters (Supplemental Table S6). Similarly, no significant interactions emerged when comparing different exercise modalities against control in interaction models with *FST* SNPs in the overall population (Supplemental Table S7). However, subgroup analysis focusing on the Hill intervention group revealed a significant genotype-exercise interaction in women: women carrying the rs3797296 G allele (AG/GG genotypes) showed significantly smaller increases in muscle mass compared to AA genotype carriers after the 16-week Hill intervention (β = −1.126, 95% CI: −1.767 to −0.485, *P*__adj_ = 0.034) (Table 5). No significant genotype-exercise interactions for muscle mass or other parameters were observed within the Combined, Running, or Cycling groups when analyzed separately (Supplemental Figure S1).

### 3.5. Follistatin Analysis: Baseline Associations, Exercise-Induced Changes, and Mediation

**Figure 3:**
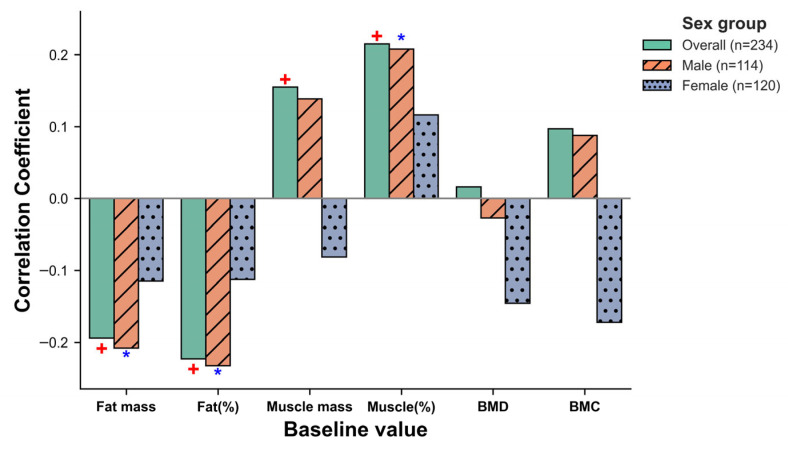
Correlations of baseline follistatin with baseline body composition and bone mineral parameters by sex. This figure presents Spearman’s rank correlation coefficients between baseline follistatin and baseline body composition/bone mineral parameters for the overall sample (solid green bars; n = 234), men (orange bars with diagonal hatch fill; n = 114), and women (blue bars with dot pattern fill; n = 120). Because the data were non-normally distributed, Spearman’s rank correlation was used. Blue asterisks (*: *p* < 0.05) indicate significant correlations prior to multiple comparison correction, while red plus signs (+: *P*__adj_ < 0.05) denote significance following Benjamini–Hochberg adjustment.

Analyses of follistatin levels and their changes were conducted in the subset of 234 participants for whom serum follistatin data were available. Baseline serum follistatin levels were negatively correlated with baseline fat mass and fat percentage, and positively with muscle mass and muscle percentage in the overall sample (Figure 3). Stratified analyses showed these correlations were more pronounced and statistically significant in men but not in women. Baseline follistatin levels were also higher in men (Supplemental Table S8). Exercise intervention led to a significant increase in follistatin levels in the pooled exercise group overall and in men, particularly in the Hill and Running groups (Supplemental Table S9). Mediation analyses did not support changes in follistatin as a significant mediator of the relationships between *FST* SNPs and exercise-induced changes in body composition or bone mineral parameters (Supplemental Table S10).

### 3.6. In Silico Functional Characterization of rs3797296 and rs3797297

Functional annotation of the two independent SNPs revealed distinct profiles. PheWAS analysis using UK Biobank data provided strong evidence for the functional relevance of rs3797297. It exhibited highly significant associations with multiple musculoskeletal traits, most notably with appendicular lean mass (*p* = 1.0 × 10^−20^), trunk fat-free mass (*p* = 1.8 × 10^−8^), and standing height (*p* = 2.0 × 10^−9^). In stark contrast, no significant associations were found for rs3797296 in the same database.

To explore a potential mechanism for rs3797297, we queried the GTEx portal. In skeletal muscle, this SNP was identified as a significant expression quantitative trait locus (eQTL) for the gene *NDUFS4* (NADH: Ubiquinone Oxidoreductase Core Subunit S4) (*p* = 1.59 × 10^−4^). The T allele correlated with a dose-dependent increase in *NDUFS4* expression (Normalized Effect Size = 0.074). No significant eQTLs were identified for rs3797296 in skeletal muscle.

Epigenetic data further supported a regulatory role for rs3797297, which resides in a region marked by active enhancer signatures and received a high regulatory potential ranking from RegulomeDB (Rank: 1f). In comparison, rs3797296 showed weaker evidence of regulatory function (Rank: 4).

## 4. Discussion

This study provides novel insights into the role of *FST* gene polymorphisms (rs3797296, rs3797297) in relation to baseline body composition, bone mineral status, and responses to different exercise modalities over 16 weeks in a Northern Han Chinese population. Key findings include a sex-specific association of the rs3797297 T allele with greater baseline muscle mass and muscle-mediated BMC in women, and a gene-exercise interaction where the rs3797296 G allele attenuated muscle mass gains in women undertaking Hill climbing. While exercise improved body composition, particularly in women, and increased serum follistatin mainly in men after Hill/Running, follistatin did not mediate the observed *FST* SNP effects on exercise adaptation. These results highlight complex interactions between *FST* genetics, sex, exercise modality, and musculoskeletal outcomes.

The observed positive association between the *FST* rs3797297 T allele and higher baseline muscle mass exclusively in women suggests a potential sex-specific role for this variant in muscle regulation within this population. Follistatin, encoded by *FST*, is a critical negative regulator of TGF-β superfamily members like myostatin and activin A, which inhibit muscle growth [22,42]. Genetic variations within *FST* could potentially alter follistatin’s structure, expression, or binding affinity, thereby influencing downstream signaling pathways (Smad2/3 vs. Smad1/5/8 balance) and ultimately muscle phenotype [23]. While direct functional evidence for rs3797297 is lacking, our finding aligns conceptually with studies linking other *FST* variants (e.g., rs722910) to strength indices [43] and numerous reports correlating higher circulating follistatin levels with greater lean body mass or muscle mass [44,45]. Interestingly, while we also observed a positive correlation between baseline follistatin levels and muscle mass/percentage, this correlation was statistically significant and more pronounced in men, contrasting with the SNP association found only in women. This discrepancy underscores potential sex-specific mechanisms perhaps involving hormonal influences or differential roles of genetic variants versus circulating protein levels.

Furthermore, the rs3797297 T allele was associated with higher baseline BMC in women, an effect significantly mediated by muscle mass according to our analysis. This finding underscores the well-established mechanical and endocrine crosstalk between muscle and bone [46,47,48]. Higher muscle mass exerts greater mechanical loading on bone and secretes myokines that can influence bone metabolism, thus contributing to increased bone mineral content [46,47]. Our data suggest this *FST* variant influences female BMC primarily via muscle, not direct bone effects. Inconsistent reports on follistatin-bone links [49,50] and our null correlation further highlight the complexity of bone regulation beyond single factors. Our data nominate rs3797297 as a potential genetic marker influencing female bone mineral status indirectly through modulation of muscle mass.

We found no significant *FST* SNPs associations with baseline fat mass, despite preclinical evidence for follistatin’s role in adipose tissue [25,51] and our observation of an inverse correlation between baseline follistatin levels and fat mass (stronger in men). Given follistatin’s function in regulating TGF-β ligands which can influence adipogenesis and inflammation [20], its relationship with fat metabolism is likely complex and potentially indirect or context-dependent. The discrepancy between SNP associations (null) and follistatin level correlations (significant in men) warrants further investigation in larger cohorts to elucidate the nuanced interplay between *FST* genetics, circulating follistatin, and adipose tissue regulation.

Consistent with the extensive literature [52,53], our 16-week structured exercise interventions significantly improved body composition compared to controls. Women showed broad improvements across all modalities, potentially due to higher baseline fat allowing larger relative changes [54], while men responded significantly primarily in Combined and Hill groups. Notably, only Hill climbing significantly increased BMC (in women/overall), possibly due to unique loading patterns (e.g., eccentric work). However, this interpretation must be tempered by the significant limitation of non-standardized exercise doses across groups; the longer duration single session of the Hill group cannot be directly compared in efficacy to the shorter, more frequent sessions of other groups based on these data.

A key finding was the rs3797296 G allele associated with attenuated Hill-induced muscle gains in women. This suggests that *FST* genetic variation can modulate adaptation to specific types of exercise in a sex-specific manner. Our result contrasts with Kostek et al. [43], who found no interaction between other *FST* SNPs and resistance training responses in a different population, highlighting that the functional impact of *FST* variants may depend on the specific polymorphism, exercise modality characteristics (e.g., aerobic vs. resistance, eccentric component), and population background. We also observed increased serum follistatin post-exercise (overall and in men, especially Hill/Running), consistent with some studies linking it to exercise stress or muscle recruitment [55,56,57]. The lack of significant increase in women might relate to lower muscle mass or other factors. However, given our null mediation results, changes in circulating follistatin do not appear to be the primary mechanism explaining the observed interaction effect of the rs3797296 genotype on muscle mass gains in women undertaking the Hill climbing intervention.

A key objective was to explore serum follistatin as a potential mediator linking *FST* SNPs to exercise-induced adaptations. However, despite observing baseline correlations between follistatin and body composition metrics, and exercise-induced increases in follistatin (primarily in men), our formal mediation analyses did not support circulating follistatin as a significant mediator of the identified *FST* SNP effects on changes in muscle mass or BMC following the interventions. Potential reasons include limited power in the follistatin subset (n = 234), the possibility that circulating levels do not reflect local tissue actions or binding dynamics, and the inherent complexity of *FST* signaling pathways [20,22,42] likely involving multiple mediators beyond just serum follistatin. Thus, while follistatin remains biologically relevant, it does not appear to be the primary intermediary linking *FST* genetics to exercise adaptation variability in this context.

A key strength of our study is the in-depth functional annotation, which identifies rs3797297 as the likely causal variant and reveals distinct roles for the two independent SNPs. Compelling evidence for rs3797297’s role comes from large-scale PheWAS data, where it is robustly associated with muscle mass and body composition, thus validating our cohort’s findings.

Mechanistically, we propose a pathway complementary to *FST*’s known function. The rs3797297 T allele, which we found associated with higher baseline muscle mass, significantly increases the expression of *NDUFS4* in skeletal muscle, as evidenced by GTEx eQTL data. Since *NDUFS4* is a core subunit of the mitochondrial Complex I [58], our findings strongly suggest the variant’s beneficial effect is mediated through enhanced mitochondrial bioenergetics and efficiency [59]. This hypothesis is further supported by the SNP’s location within an active enhancer region.

In contrast, rs3797296 lacks support from baseline-state public databases. However, its association with muscle mass changes following exercise in our study raises the intriguing possibility that it functions as a “response QTL” (rQTL), whose effects are unmasked by a specific stimulus [60]. This suggests a functional divergence: rs3797297 influences baseline traits, while rs3797296 may modulate exercise adaptability.

In conclusion, our bioinformatic investigation moves beyond simple association to propose a testable hypothesis for rs3797297’s mechanism via *NDUFS4* regulation. Future experimental work, such as CRISPR-based editing, is necessary to definitively confirm these in silico findings.

Limitations should be considered. The Northern Han Chinese sample restricts generalizability, particularly to other ethnic groups or populations with different lifestyle patterns. While the overall sample size (n = 470) provided adequate power for main effect analyses, the availability of complete serum follistatin data was limited in a subset (n = 234) due to factors such as insufficient venous blood volume collected from some participants. This smaller subsample significantly reduced statistical power for follistatin-related analyses, including correlations, exercise-induced changes (especially in subgroups), and particularly the mediation models, thereby limiting the robustness of these specific findings. Another important consideration is that our SNPs genotyping was initiated in 2015, which means our findings should be interpreted in the context of scientific advancements and evolving understandings of exercise physiology and genetics since that time. The non-standardized exercise dose across modalities is a major limitation, precluding direct comparisons of efficacy between exercise types and necessitating caution when interpreting modality-specific findings like the Hill group’s BMC increase. Lack of objective dose monitoring further restricts interpretation. Unmeasured confounders (diet, sleep, daily physical activity levels, and stress) could influence results. Moreover, our study focused on a limited number of *FST* SNPs; a broader panel of genetic variants might reveal additional associations. Crucially, the absence of independent external validation cohorts represents a significant limitation; while our findings provide novel insights within the Northern Han Chinese population, their generalizability would be strengthened by replication in diverse independent populations. Finally, biological mechanisms are complex and likely extend beyond serum follistatin.

Despite these limitations, our findings contribute significantly to understanding individual variability in exercise response and lay groundwork for personalized exercise strategies. For instance, genetic screening for *FST* rs3797296 could potentially identify women who might be less responsive to muscle mass gains during hill climbing interventions, guiding personalized adjustments to their training programs. This study thus underscores the broader potential of precision medicine in exercise science, moving beyond ‘one-size-fits-all’ recommendations towards genetically informed exercise prescriptions. Future research needs larger, diverse samples, standardized/monitored exercise protocols allowing dose–response analysis, control for confounding factors, and multi-omics approaches to elucidate mechanisms and validate these exploratory findings for potential personalized exercise applications.

Specifically, future studies should focus on the following:

(1) recruiting broader and more diverse cohorts to enhance generalizability and provide independent external validation of genetic associations;
(2) implementing precisely standardized exercise interventions with objective dose monitoring to enable direct efficacy comparisons and dose–response modeling;
(3) comprehensively assessing and controlling for a wider array of confounding factors like diet, sleep, daily physical activity levels, and stress; and
(4) integrating multi-omics data (e.g., epigenomics, transcriptomics, proteomics) alongside genetic information to gain deeper insights into the complex biological pathways underlying gene–exercise interactions. Ultimately, validating these preliminary findings in larger, independent studies is essential to translate genetic insights into practical, personalized exercise recommendations for optimizing musculoskeletal health. It is crucial to note that while genetic insights can refine individual exercise strategies, regular physical activity remains a cornerstone for musculoskeletal health for everyone, irrespective of their genetic predisposition.

## 5. Conclusions

In conclusion, within this Northern Han Chinese population, the *FST* rs3797297 T allele associates with greater muscle mass and muscle-mediated BMC specifically in women. Importantly, functional annotation suggests this effect is mediated by increased expression of the mitochondrial gene *NDUFS4*, highlighting a novel link between *FST* genetics and muscle bioenergetics. Furthermore, the rs3797296 G allele was linked to blunted muscle mass adaptation uniquely in women undergoing hill-climbing exercise, highlighting that genetic influences on exercise outcomes are dependent on both sex and exercise modality. While baseline serum follistatin correlated with body composition and increased post-exercise (primarily in men), it did not emerge as a significant mediator for the observed SNP-exercise adaptation relationships. Collectively, these findings contribute to understanding exercise response variability and underscore the potential for genetically informed exercise prescription. However, given study limitations, particularly the non-standardized exercise dose, validation in larger, diverse populations using standardized protocols is crucial. Future multi-omics approaches are also needed to fully elucidate the molecular mechanisms underlying these SNP-phenotype associations and to explore their translational applications.

## Figures and Tables

**Table 1:** Basic characteristics of Chinese participants.

	Total (n = 470)	Male (n = 208)	Female (n = 262)
Age (years) ^a^	37 (23–50)	33.5 (22–49)	40 (24–50)
Height (cm) ^b^	165.68 ± 7.95	172.14 ± 5.63	160.55 ± 5.36 ***
Weight (kg) ^b^	63.82 ± 11.36	71.31 ± 10.60	57.88 ± 7.92 ***
BMI (kg/m^2^) ^b^	23.21 ± 3.25	24.06 ± 3.35	22.53 ± 3.01 ***
Waist circumference ^b^	81.32 ± 9.94	84.64 ± 9.95	78.67 ± 9.12 ***
Hip circumference ^b^	93.42 ± 5.85	94.18 ± 6.10	92.81 ± 5.58 *
Waist-Hip ratio ^b^	0.87 ± 0.07	0.90 ± 0.07	0.85 ± 0.07 ***
Fat mass (kg)	17.22 (12.43–22.00)	15.66 (9.15–20.99)	17.92 (14.43–22.93) ^###^
Fat (%)	27.74 (21.32–33.65)	22.41 (14.05–27.60)	31.79 (27.00–36.61) ^###^
Muscle mass (kg)	40.75 (36.17–51.44)	52.48 (48.75–56.54)	36.54 (34.30–38.77) ^###^
Muscle (%)	67.75 (62.11–74.24)	72.83 (68.47–81.40)	63.35 (58.93–68.53) ^###^
BMD (g/cm^3^)	1.15 ± 0.10	1.20 ± 0.09	1.11 ± 0.08 ***
BMC (kg)	2.56 (2.24–2.90)	2.91 (2.67–3.15)	2.33 (2.11–2.55) ^###^

^a^ Data are non-normally distributed and presented as median (interquartile range, IQR). ^b^ Data are normally distributed and presented as mean ± SD. BMI: Body Mass Index; BMD: Bone Mineral Density; BMC: Bone Mineral Content. *p* Value indicates comparisons between males and females. For normally distributed data, an independent samples t-test was used (*: *p* < 0.05, ***: *p* < 0.001). For non-normally distributed data, a Mann–Whitney U test was applied (###: *p* < 0.001).3.2. SNP Genotyping.

**Table 2:** Basic information on SNP loci of Chinese participants.

Chromosome (Forward Strand)	ID	Sex	Allele	MAF	Genotype: Frequency (Count)		χ^2^	*P*_HWE
5: 52780353	rs12152850	Total	C/T	T: 0.020	CC: 0.960 (451)	CT: 0.040 (19)	0.002	0.964
		Male	C/T	T: 0.019	CC: 0.962 (200)	CT: 0.038 (8)	0.001	0.978
		Female	C/T	T: 0.021	CC: 0.958 (251)	CT: 0.042 (11)	0.001	0.971
5: 52777721	rs3797296	Total	A/G	G: 0.188	AA: 0.655 (308)	AG/GG: 0.345 (162)	1.922	0.166
		Male	A/G	G: 0.151	AA: 0.721 (150)	AG/GG: 0.279 (58)	0.409	0.522
		Female	A/G	G: 0.218	AA: 0.603 (158)	AG/GG: 0.397 (104)	1.770	0.183
5: 52777656	rs3797297	Total	G/T	T: 0.124	GG: 0.770 (362)	GT/TT: 0.230 (108)	0.483	0.487
		Male	G/T	T: 0.161	GG: 0.726 (151)	GT/TT: 0.274 (57)	0.386	0.535
		Female	G/T	T: 0.103	GG: 0.805 (211)	GT/TT: 0.195 (51)	0.156	0.693

Chromosomal positions are based on the GRCh37 human reference genome assembly. MAF: Minor Allele Frequency. χ^2^ and *P*_HWE: Chi-square statistic and *p*-value for Hardy–Weinberg equilibrium (HWE), obtained via chi-square (χ^2^) testing, indicating whether genotype frequencies conform to HWE expectations.

**Table 3:** Associations of rs3797297 with baseline body composition and bone mineral parameters in Chinese participants.

	Males (n = 208)					Females (n = 262)				
	rs3797297					rs3797297				
	GG (n = 151)	GT/TT (n = 57)	Beta (95% CI)	*P*	*P*__adj_	GG (n = 211)	GT/TT (n = 51)	Beta (95% CI)	*P*	*P*__adj_
Fat mass (kg)	15.28 (9.14, 20.78)	16.34 (9.52, 21.34)	−0.714 (−1.719, 0.290)	0.163	0.259	17.58 (14.01, 21.91)	21.06 (15.66, 24.28)	0.606 (−0.173, 1.384)	0.127	0.206
Fat (%)	22.42 (14.00, 27.60)	22.40 (14.66, 27.30)	−0.662 (−2.049, 0.725)	0.349	0.463	31.40 (26.57, 36.49)	33.66 (30.09, 38.00)	0.080 (−1.058, 1.218)	0.890	0.963
Muscle mass (kg)	52.54 ± 5.21	52.61 ± 5.74	0.155 (−1.257, 1.567)	0.830	0.905	36.28 ± 3.33	37.62 ± 3.62	1.159 (0.202, 2.116)	**0.018**	**0.034** †
Muscle (%)	72.55 (68.58, 81.56)	73.07 (68.36, 81.30)	0.559 (−0.858, 1.977)	0.439	0.550	63.67 (59.25, 68.68)	62.13 (57.64, 65.72)	−0.046 (−1.192, 1.100)	0.937	0.963
BMD (g/cm^3^)	1.20 ± 0.09	1.19 ± 0.07	−0.011 (−0.035, 0.014)	0.390	0.501	1.10 ± 0.08	1.13 ± 0.09	0.024 (0.001, 0.047)	**0.040**	0.074
BMC (kg)	2.94 ± 0.41	2.86 ± 0.31	−0.071 (−0.173 0.032)	0.176	0.270	2.30 ± 0.30	2.43 ± 0.34	0.127 (0.039, 0.215)	**0.005**	**0.009** †

Associations between *FST* rs3797297 (GG vs. GT/TT) and baseline body composition/bone mineral parameters were analyzed using GLM, with stratification by sex and adjustment for age and baseline BMI. BMD: Bone Mineral Density; BMC: Bone Mineral Content. Beta (95% CI): Regression coefficient and 95% confidence interval. Bolded *p* values (<0.05) represent significant findings before multiple comparison correction. *P*__adj_: Benjamini–Hochberg–adjusted *p* value (<0.05 indicates statistical significance; denoted by †).

**Table 4:** Mediation of muscle mass in the relationship between rs3797297 and BMC (kg) in women.

Mediation Effect	Beta (95% CI)	95% CI Lower	95% CI Upper	*p*-Value
Total effect	0.106	0.016	0.198	**0.021**
Indirect effect	0.056	0.005	0.113	**0.043**
Direct effect	0.050	−0.023	0.123	0.181

Mediation models were adjusted for age and baseline BMI. Beta (95% CI): Regression coefficient and 95% confidence interval. Total Effect: The overall effect of the rs3797297 on BMC, without considering the mediator (muscle mass). Indirect Effect: The effect of the rs3797297 on BMC transmitted through muscle mass. Direct Effect: The effect of the rs3797297 on BMC after controlling for muscle mass. Bolded *p* values (<0.05) indicate significant findings.

**Table 5:** Associations of *FST* rs3797296 with the 16-week hill exercise intervention responses by sex in Chinese participants.

	Males			Females		
	AA (n = 29)	AG/GG (n = 10)		AA (n = 17)	AG/GG (n = 8)	
	Beta (95% CI)	*P*	*P*__adj_	Beta (95% CI)	*P*	*P*__adj_
Fat mass (kg)	0.173 (−0.807, 1.153)	0.729	0.921	−0.284 (−1.469, 0.900)	0.638	0.868
Fat (%)	0.038 (−1.013, 1.089)	0.944	0.980	0.301 (−1.083, 1.685)	0.670	0.886
Muscle mass (kg)	0.161 (−0.729, 1.052)	0.723	0.921	−1.126 (−1.767, −0.485)	**0.001**	**0.034** †
Muscle (%)	−0.213 (−1.658, 1.233)	0.773	0.938	−0.451 (−2.025, 1.123)	0.575	0.844
BMD (g/cm^3^)	−0.008 (−0.027, 0.012)	0.442	0.785	−0.010 (−0.025, 0.005)	0.180	0.658
BMC (kg)	0.015 (−0.030, 0.060)	0.523	0.833	0.014 (−0.038, 0.066)	0.594	0.854

GLM analyses were used to assess whether *FST* rs3797296 modifies the effects of the hill intervention on body composition and bone mineral parameters, adjusting for age, baseline BMI, and respective baseline values. BMD: Bone Mineral Density; BMC: Bone Mineral Content. Beta (95% CI): Regression coefficient and 95% confidence interval. Bolded *p* values (<0.05) represent significant findings before multiple comparison correction. *P_adj*: Benjamini–Hochberg–adjusted p value (<0.05 indicates statistical significance; denoted by †).

## Data Availability

All data generated or analyzed during this study are included in this published article and its supplementary information files.

## Supplementary Materials

The following supporting information can be downloaded at: https://www.mdpi.com/article/10.3390/genes16070810/s1, Figure S1: Associations Between *FST* rs3797296/rs3797297 and Exercise Response Across Different Exercise Modalities; Table S1: Associations of rs3797296/rs3797297 with Baseline Body Composition and Bone Mineral Parameters in the Total Sample; Table S2: Associations of rs3797296 with Baseline Body Composition and Bone Mineral Parameters; Table S3: Association between *FST* rs3797297 and BMC in Women; Table S4: Effects of 16-Week Exercise Intervention on Body Composition and Bone Mineral Parameters; Table S5: Effects of 16-Week Exercise Intervention on Body Composition and Bone Mineral Parameters Across Different Exercise Modalities; Table S6: Associations of *FST* rs3797296/rs3797297 with the 16-Week Exercise-Induced Changes in Body Composition and Bone Mineral Parameters; Table S7: Interactions between *FST* rs3797296/rs3797297 and Different Exercise Modalities on 16-Week Changes in Body Composition and Bone Mineral Parameters; Table S8: Baseline Serum Follistatin Levels by Sex and Group; Table S9: Changes in Serum Follistatin Following 16-Week Exercise Intervention by Exercise Modality and Sex; Table S10 Mediation Analysis of Follistatin Changes in the Relationship Between *FST* rs3797296/rs3797297 and Exercise Response; Table S11: Mediation Analysis of Follistatin Changes in the Relationship Between *FST* rs3797296/rs3797297 and Exercise Response by Exercise Modalities.
