# Supplementary material for: FST Polymorphisms Associate with Musculoskeletal Traits and Modulate Exercise Response Differentially by Sex and Modality in Northern Han Chinese Adults
Source: Genes (Basel). 2025 Jul 10;16(7):810. doi: 10.3390/genes16070810 (PMC12295022; doi:10.3390/genes16070810)
Supplement: Supplementary file 1 [file genes-16-00810-s001.zip › genes-3733837-supplementary.pdf]

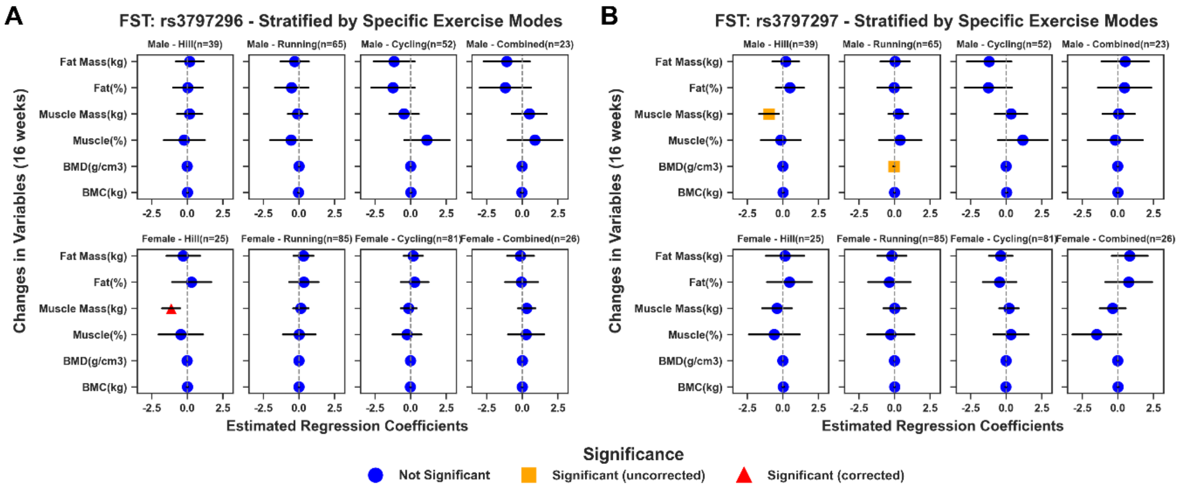


**Supplement Figure S1. Associations Between *FST* rs3797296/rs3797297 and Exercise Response Across Different Exercise Modalities**

This figure presents the results of association analyses between two FST gene loci (**Fig. A**: rs3797296; **Fig. B**: rs3797297) and changes in body composition and bone mineral parameters following 16 weeks of different exercise modalities. GLM were used, with 16-week changes in body composition and bone mineral parameters as the dependent variable, FST rs3797296/rs3797297 as the independent variables, and adjustments for baseline values, age, and baseline BMI as covariates. Dots and error bars represent regression coefficient estimates and their 95% confidence intervals, color-coded to indicate statistical significance (blue circles, nonsignificant; orange squares, significant before adjustment; red triangles, significant after Benjamini-Hochberg correction).

**Supplement Table S1. Associations of rs3797296/rs3797297 with Baseline Body Composition and Bone Mineral Parameters in the Total Sample**

| | **rs3797296** | | **Beta (95% CI)** | ***P*** | ***Adj_P*** | **Sex Effect** | | |
|---|---|---|---|---|---|---|---|---|
| | **AA (n=308)** | **AG/GG (n=162)** | | | | **Beta (95%CI)** | ***P*** | ***Adj_P*** |
| **Fat mass(kg)** | 17.25±7.05 | 17.48±7.01 | 0.10 | 0.717 | 0.796 | 5.81 | **3.2E-92** | **8.8E-92**┼ |
| | | | (-0.46, 0.67) | | | (5.25, 6.37) | | |
| **Fat(%)** | 27.00 | 29.38 | 0.10 | 0.797 | 0.854 | 13.06 | **7.6E-236** | **3.8E-235**┼ |
| | (20.72, 32.81) | (22.30, 34.18) | (-0.68, 0.89) | | | (12.28, 13.85) | | |
| **Muscle mass(kg)** | 42.37 | 38.61 | -0.04 | 0.907 | 0.914 | -14.74 | **0.0E+00** | **0.0E+00**┼ |
| | (36.54, 52.20) | (35.47, 49.12) | (-0.79, 0.70) | | | (-15.48, -14.00) | | |
| **Muscle(%)** | 68.48 | 66.42 | -0.06 | 0.891 | 0.914 | -13.44 | **7.1E-242** | **4.3E-241**┼ |
| | (62.64, 74.67) | (61.35, 72.58) | (-0.85, 0.74) | | | (-14.23, -12.65) | | |

| | rs3797297 | | Beta (95%CI) | *P* | *Adj_P* | Sex Effect | | |
|---|---|---|---|---|---|---|---|---|
| | **GG (n=362)** | **GT/TT (n=108)** | | | | **Beta (95%CI)** | ***P*** | ***Adj_P*** |
| **BMD(g/cm$^3$)** | 1.16±0.10 | 1.13±0.09 | -0.01 | 0.176 | 0.216 | -0.07 | **1.5E-18** | **2.6E-18**┼ |
| | | | (-0.03, 0.01) | | | (-0.08, -0.05) | | |
| **BMC(kg)** | 2.62 | 2.45 | -0.01 | 0.837 | 0.881 | -0.50 | **6.0E-60** | **1.3E-59**┼ |
| | (2.27, 2.92) | (2.21, 2.82) | (-0.07 0.05) | | | (-0.56, -0.44) | | |
| **Fat mass(kg)** | 17.17±7.00 | 17.85±7.15 | -0.03 | 0.914 | 0.91 | 5.82 | **7.3E-93** | **2.1E-92**┼ |
| | | | (-0.67, 0.60) | | | (5.26, 6.38) | | |
| **Fat(%)** | 27.74 | 27.65 | -0.27 | 0.553 | 0.65 | 13.06 | **1.0E-236** | **5.5E-236**┼ |
| | (21.49, 33.62) | (20.44, 33.69) | (-1.15, 0.62) | | | (12.28, 13.84) | | |
| **Muscle mass(kg)** | 39.71 | 44.68 | 0.55 | 0.200 | 0.24 | -14.70 | **0.0E+00** | **0.0E+00**┼ |
| | (35.80, 50.85) | (37.62, 52.98) | (-0.29, 1.38) | | | (-15.44, -13.97) | | |

| | | | | | | | | |
|---|---|---|---|---|---|---|---|---|
| **Muscle(%)** | 67.57 | 68.30 | 0.25 | 0.581 | 0.67 | -13.43 | **1.2E-242** | **7.9E-242**┼ |
| | (62.05, 74.03) | (62.58, 74.90) | (-0.64, 1.15) | | | (-14.22, -12.64) | | |
| **BMD(g/cm$^3$)** | 1.14 | 1.17 | 0.003 | 0.699 | 0.79 | -0.07 | **5.2E-19** | **9.5E-19**┼ |
| | (1.08, 1.20) | (1.11, 1.22) | (-0.01, 0.02) | | | (-0.08, -0.05) | | |
| **BMC(kg)** | 2.51 | 2.71 | 0.01 | 0.784 | 0.85 | -0.50 | **3.3E-60** | **7.4E-60** |
| | (2.22, 2.90) | (2.36, 2.90) | (-0.06, 0.08) | | | (-0.56, -0.44) | | |

Associations between FST rs3797296/rs3797297 with baseline body composition/bone mineral parameters in the total sample were analyzed using generalized linear models (GLM), adjusted for age and baseline BMI.

**Beta (95% CI):** Regression coefficient and 95% confidence interval.

Bolded p values (< 0.05) represent significant findings before multiple comparison correction.

***Adj_p*:** Benjamini-Hochberg – adjusted p value (< 0.05 indicates statistical significance; denoted by ┼).

**Supplement Table S2. Associations of rs3797296 with Baseline Body Composition and Bone Mineral Parameters**

| | Males(n=208) | | | | | Females(n=262) | | | | |
|---|---|---|---|---|---|---|---|---|---|---|
| | rs3797296 | | Beta (95% CI) | *P* | *Adj_P* | rs3797296 | | Beta (95%CI) | *P* | *Adj_P* |
| | AA (n=150) | AG/GG (n=58) | | | | AA (n=158) | AG/GG (n=104) | | | |
| **Fat mass(kg)** | 15.81 (9.39, 20.79) | 14.84 (8.84, 21.35) | 0.13 (-0.86, 1.13) | 0.794 | 0.873 | 18.71 ± 6.07 | 18.48 ± 5.90 | 0.11 (-0.52, 0.73) | 0.736 | 0.845 |
| **Fat(%)** | 22.51 (14.73, 26.98) | 22.11 (13.04, 28.73) | -0.08 (-1.45, 1.29) | 0.911 | 0.963 | 31.47 ± 6.65 | 31.50 ± 6.93 | 0.27 (-0.64, 1.18) | 0.563 | 0.675 |
| **Muscle mass(kg)** | 52.56 ± 5.24 | 52.57 ± 5.65 | 0.04 (-1.35, 1.44) | 0.950 | 0.963 | 36.70 ± 3.48 | 36.30 ± 3.33 | -0.24 (-1.02, 0.53) | 0.536 | 0.665 |
| **Muscle(%)** | 72.64 (68.68, 81.07) | 73.06 (67.33, 82.82) | 0.25 (-1.15, 1.65) | 0.729 | 0.845 | 63.79 ± 6.39 | 63.77 ± 6.73 | -0.27 (-1.19, 0.64) | 0.559 | 0.675 |

| | | | | | | | | | | |
|---|---|---|---|---|---|---|---|---|---|---|
| **BMD(g/cm$^3$)** | 1.20 ± 0.09 | 1.19 ± 0.08 | -0.01 | 0.546 | 0.671 | 1.12 ± 0.08 | 1.10 ± 0.08 | -0.01 | 0.213 | 0.310 |
| | | | (-0.03, 0.02) | | | | | (-0.03, 0.01) | | |
| **BMC(kg)** | 2.92 ± 0.37 | 2.93 ± 0.41 | 0.02 | 0.769 | 0.858 | 2.34 ± 0.33 | 2.30 ± 0.28 | -0.03 | 0.412 | 0.521 |
| | | | (-0.09, 0.12) | | | | | (-0.10, 0.04) | | |

Associations between FST rs3797296 (AA vs. AG/GG) and baseline body composition/bone mineral parameters were analyzed using GLM, with stratification by sex and adjustment for age and baseline BMI.

**Beta (95% CI):** Regression coefficient and 95% confidence interval.

**Bolded p values** (< 0.05) represent significant findings before multiple comparison correction.

***Adj_p*:** Benjamini-Hochberg ‑ adjusted p value (< 0.05 indicates statistical significance; denoted by ┼).

**Supplement Table S3. Association between *FST* rs3797297 and BMC in Women**

| BMC(kg) | Beta | 95% CI lower | 95% CI upper | *P* | *Adj_P* |
|---|---|---|---|---|---|
| **Model_1** | 0.11 | 0.02 | 0.19 | **0.012** | **0.025┼** |
| **Model_2** | 0.07 | -0.01 | 0.14 | 0.075 | 0.116 |

Associations between rs3797297 and BMC in women were analyzed using GLM, and adjustment for age and baseline BMI. In Model 1, fat mass was added as an additional covariate, while Model 2 included muscle mass as an additional covariate.

Bolded p values (< 0.05) represent significant findings before multiple comparison correction.

***Adj_p*:** Benjamini-Hochberg ‑ adjusted p value (< 0.05 indicates statistical significance; denoted by ┼).

**Supplement Table S4. Effects of 16-Week Exercise Intervention on Body Composition and Bone Mineral Parameters**

| | Sex | N | Ages | Baseline | Week16 | Δ | *P* |
|---|---|---|---|---|---|---|---|
| **Training group** | | | | | | | |
| **Weight(kg)** | Total | 396 | 37 (23, 49) | 62.15 (55.80, 70.43) | 61.90 (55.83, 70.65) | -0.20 (-1.30, 0.80) | **0.003** |
| | Male | 179 | 34 (22, 48) | 71.27 ± 10.47 | 71.17 ± 10.27 | 0.00 (-1.20, 1.00) | 0.530 |
| | Female | 217 | 40 (23, 50) | 57.69 ± 7.42 | 57.19 ± 7.23 | -0.40 (-1.40, 0.60) | **0.000** |
| **Fat mass(kg)** | Total | 396 | 37 (23, 49) | 17.13 ± 6.95 | 16.79 ± 6.67 | -0.23 (-1.38, 0.71) | **0.000** |
| | Male | 179 | 34 (22, 48) | 15.64 (9.07, 21.10) | 15.61 (9.83, 21.70) | 0.04 (-1.13, 0.95) | 0.804 |
| | Female | 217 | 40 (23, 50) | 17.81 (14.26, 22.27) | 17.43 (14.09, 21.55) | -0.53 (-1.53, 0.47) | **0.000** |
| **Fat(%)** | Total | 396 | 37 (23, 49) | 27.37 (21.01, 33.11) | 27.62 (20.82, 32.78) | -0.29 (-1.68, 0.95) | **0.001** |
| | Male | 179 | 34 (22, 48) | 22.20 (14.28, 27.75) | 22.23 (14.83, 27.91) | -0.09 (-1.12, 1.27) | 0.885 |
| | Female | 217 | 40 (23, 50) | 31.17 ± 6.77 | 30.49 ± 6.72 | -0.68 ± 2.19 | **0.000** |
| **Muscle mass(kg)** | Total | 396 | 37 (23, 49) | 40.69 (36.25, 51.90) | 41.16 (36.29, 52.09) | 0.01 (-0.76, 0.87) | 0.363 |

| | | | | | | | |
|---|---|---|---|---|---|---|---|
| | Male | 179 | 34 (22, 48) | 52.57 ± 5.38 | 52.62 ± 5.36 | 0.09 ± 1.45 | 0.677 |
| | Female | 217 | 40 (23, 50) | 36.63 ± 3.32 | 36.78 ± 3.34 | 0.15 ± 1.21 | 0.070 |
| **Muscle(%)** | Total | 396 | 37 (23, 49) | 68.23 (62.42, 74.56) | 68.20 (63.00, 75.21) | 0.50 (-1.15, 1.91) | **0.000** |
| | Male | 179 | 34 (22, 48) | 72.92 (68.30, 81.23) | 73.66 (68.64, 81.28) | 0.19 ± 2.54 | 0.542 |
| | Female | 217 | 40 (23, 50) | 64.08 ± 6.55 | 64.35 (60.25, 68.93) | 0.80 ± 2.36 | **0.000** |
| **BMD(g/cm$^3$)** | Total | 396 | 37 (23, 49) | 1.16 ± 0.10 | 1.15 ± 0.10 | -0.00 (-0.01, 0.01) | 0.205 |
| | Male | 179 | 34 (22, 48) | 1.21 ± 0.09 | 1.20 (1.14, 1.27) | -0.00 (-0.01, 0.01) | 0.599 |
| | Female | 217 | 40 (23, 50) | 1.12 (1.06, 1.18) | 1.12 (1.06, 1.17) | -0.00 (-0.01, 0.01) | 0.305 |
| **BMC(kg)** | Total | 396 | 37 (23, 49) | 2.59 (2.27, 2.93) | 2.58 (2.28, 2.93) | -0.00 (-0.04, 0.03) | 0.173 |
| | Male | 179 | 34 (22, 48) | 2.94 ± 0.39 | 2.94 ± 0.39 | -0.00 (-0.04, 0.04) | 0.952 |
| | Female | 217 | 40 (23, 50) | 2.35 ± 0.32 | 2.34 ± 0.32 | -0.00 (-0.04, 0.03) | 0.094 |
| **Control group** | | | | | | | |
| **Weight(kg)** | Total | 74 | 39 (26, 54.8) | 63.79 ± 12.30 | 64.73 ± 12.45 | 0.94 ± 1.80 | **0.000** |

| | | | | | | | |
|---|---|---|---|---|---|---|---|
| | Male | 29 | 32 (25, 59) | 71.56 ± 11.59 | 72.42 ± 12.10 | 0.87 ± 1.70 | **0.011** |
| | Female | 45 | 43 (30, 54) | 58.79 ± 10.01 | 59.78 ± 10.01 | 0.98 ± 1.87 | **0.001** |
| **Fat mass(kg)** | Total | 74 | 39 (26, 54.8) | 18.38 ± 7.41 | 19.10 ± 7.42 | 0.71 ± 1.40 | **0.000** |
| | Male | 29 | 32 (25, 59) | 15.98 ± 8.06 | 16.62 ± 8.13 | 0.64 ± 1.16 | **0.006** |
| | Female | 45 | 43 (30, 54) | 19.93 ± 6.60 | 20.69 ± 6.53 | 0.76 ± 1.54 | **0.002** |
| **Fat(%)** | Total | 74 | 39 (26, 54.8) | 30.00 (22.45, 35.25) | 31.30 (22.88, 35.42) | 0.60 (-0.40, 1.75) | **0.005** |
| | Male | 29 | 32 (25, 59) | 21.27 ± 8.20 | 21.84 ± 8.06 | 0.58 ± 1.48 | **0.044** |
| | Female | 45 | 43 (30, 54) | 32.98 ± 6.53 | 33.81 ± 6.18 | 0.83 ± 2.24 | **0.017** |
| **Muscle mass(kg)** | Total | 74 | 39 (26, 54.8) | 40.99 (34.79, 49.91) | 40.43 (35.20, 50.91) | 0.21 (-0.61, 1.06) | 0.068 |
| | Male | 29 | 32 (25, 59) | 52.49 ± 5.22 | 52.57 ± 5.06 | 0.08 ± 1.49 | 0.785 |
| | Female | 45 | 43 (30, 54) | 36.13 ± 3.88 | 36.36 ± 4.12 | 0.22 ± 1.26 | 0.240 |
| **Muscle(%)** | Total | 74 | 39 (26, 54.8) | 64.96 (60.28, 72.99) | 64.24 (60.32, 72.50) | -0.75 ± 2.10 | **0.009** |
| | Male | 29 | 32 (25, 59) | 74.37 ± 7.92 | 73.66 ± 7.76 | -0.71 ± 2.04 | 0.072 |

| | | | | | | | |
|---|---|---|---|---|---|---|---|
| | Female | 45 | 43 (30, 54) | 61.85 (57.09, 65.01) | 61.54 ± 5.60 | -0.77 ± 2.16 | **0.035** |
| **BMD(g/cm$^3$)** | Total | 74 | 39 (26, 54.8) | 1.11 ± 0.08 | 1.11 ± 0.08 | 0.00 (-0.01, 0.01) | 0.164 |
| | Male | 29 | 32 (25, 59) | 1.14 ± 0.07 | 1.15 ± 0.08 | 0.00 ± 0.02 | 0.154 |
| | Female | 45 | 43 (30, 54) | 1.08 ± 0.07 | 1.09 ± 0.07 | 0.00 (-0.01, 0.01) | 0.511 |
| **BMC(kg)** | Total | 74 | 39 (26, 54.8) | 2.44 ± 0.39 | 2.45 ± 0.40 | 0.01 ± 0.07 | 0.275 |
| | Male | 29 | 32 (25, 59) | 2.78 ± 0.30 | 2.79 ± 0.30 | 0.01 ± 0.07 | 0.493 |
| | Female | 45 | 43 (30, 54) | 2.23 ± 0.28 | 2.24 ± 0.28 | 0.01 ± 0.07 | 0.404 |

Data are presented as mean ± SD or median (IQR), depending on the distribution. Comparisons between baseline and 16-week follow-up values were conducted using paired t-tests (for normally distributed data) or Wilcoxon signed-rank tests (for non-normally distributed data).

**Training group** refers to the group that underwent the 16-week exercise intervention, while the **Control group** refers to the non-intervention group.

Δ represents the difference between Week 16 and Baseline (Week 16 – Baseline).

**Bolded P values** indicate statistical significance ($P < 0.05$).

**Supplement Table S5. Effects of 16-Week Exercise Intervention on Body Composition and Bone Mineral Parameters Across Different Exercise Modalities**

| | Sex | N | Ages | Baseline | Week16 | Δ | *P* |
|---|---|---|---|---|---|---|---|
| **Hill group** | | | | | | | |
| **Weight(kg)** | Total | 64 | 45.5 (33, 54) | 68.20 ± 11.52 | 67.61 ± 11.12 | -0.40 (-1.20, 0.60) | **0.015** |
| | Male | 39 | 45 (31, 49.5) | 73.15 ± 10.55 | 72.45 ± 9.94 | -0.70 (-1.60, 0.35) | **0.031** |
| | Female | 25 | 49 (40, 54) | 60.49 ± 8.37 | 60.07 ± 8.41 | -0.30 (-1.10, 0.60) | 0.266 |
| **Fat mass(kg)** | Total | 64 | 45.5 (33, 54) | 18.71 ± 6.83 | 18.20 ± 6.53 | -0.25 (-1.24, 0.37) | **0.008** |
| | Male | 39 | 45 (31, 49.5) | 17.77 ± 7.08 | 17.30 (14.63, 22.56) | -0.49 ± 1.50 | 0.093 |
| | Female | 25 | 49 (40, 54) | 20.19 ± 6.29 | 19.64 ± 6.32 | -0.29 (-1.13, 0.42) | 0.092 |
| **Fat(%)** | Total | 64 | 45.5 (33, 54) | 27.08 ± 8.50 | 26.59 ± 8.38 | -0.48 ± 1.62 | **0.020** |
| | Male | 39 | 45 (31, 49.5) | 24.70 (20.45, 28.75) | 25.00 (20.60, 27.80) | -0.35 ± 1.51 | 0.215 |
| | Female | 25 | 49 (40, 54) | 32.20 (31.10, 37.70) | 33.40 (28.10, 37.20) | -0.69 ± 1.80 | 0.113 |

| | | | | | | | |
|---|---|---|---|---|---|---|---|
| **Muscle mass(kg)** | Total | 64 | 45.5 (33, 54) | 48.05 (38.74, 54.56) | 48.27 (40.05, 54.40) | 0.28 ± 1.01 | **0.036** |
| | Male | 39 | 45 (31, 49.5) | 52.88 ± 4.80 | 53.05 ± 4.75 | 0.17 ± 1.03 | 0.314 |
| | Female | 25 | 49 (40, 54) | 37.91 ± 3.77 | 38.36 ± 4.10 | 0.44 ± 0.96 | **0.030** |
| **Muscle(%)** | Total | 64 | 45.5 (33, 54) | 69.30 ± 8.73 | 70.27 ± 8.55 | 0.96 ± 1.97 | **0.000** |
| | Male | 39 | 45 (31, 49.5) | 70.58 (68.28, 76.24) | 72.51 (69.51, 76.57) | 0.64 (-0.32, 1.53) | **0.017** |
| | Female | 25 | 49 (40, 54) | 62.98 (58.63, 64.84) | 63.21 (59.94, 68.05) | 1.16 ± 2.03 | **0.007** |
| **BMD($g/cm^3$)** | Total | 64 | 45.5 (33, 54) | 1.18 ± 0.10 | 1.19 ± 0.10 | 0.00 (-0.01, 0.02) | 0.341 |
| | Male | 39 | 45 (31, 49.5) | 1.22 ± 0.09 | 1.23 ± 0.09 | 0.00 (-0.01, 0.01) | 0.819 |
| | Female | 25 | 49 (40, 54) | 1.12 ± 0.09 | 1.13 ± 0.09 | 0.01 ± 0.02 | 0.144 |
| **BMC(kg)** | Total | 64 | 45.5 (33, 54) | 2.71 ± 0.49 | 2.69 ± 0.49 | -0.02 ± 0.06 | **0.024** |
| | Male | 39 | 45 (31, 49.5) | 2.94 ± 0.41 | 2.93 ± 0.40 | -0.01 ± 0.06 | 0.289 |
| | Female | 25 | 49 (40, 54) | 2.35 ± 0.38 | 2.32 ± 0.37 | -0.03 ± 0.06 | **0.029** |
| **Running group** | | | | | | | |

| | | | | | | | |
|---|---|---|---|---|---|---|---|
| **Weight(kg)** | Total | 150 | 39 (23, 50) | 61.75 (57.25, 69.78) | 61.20 (55.98, 70.50) | -0.44 ± 1.86 | **0.004** |
| | Male | 65 | 34 (22, 53) | 70.10 (63.50, 77.20) | 71.04 ± 9.28 | -0.09 ± 1.96 | 0.589 |
| | Female | 85 | 41 (24, 49) | 57.59 ± 7.28 | 56.89 ± 7.20 | -0.70 ± 1.75 | **0.000** |
| **Fat mass(kg)** | Total | 150 | 39 (23, 50) | 16.94 ± 6.71 | 16.57 ± 6.62 | -0.39 (-1.49, 0.57) | **0.012** |
| | Male | 65 | 34 (22, 53) | 15.54 ± 7.35 | 15.47 ± 7.38 | -0.14 (-1.00, 0.85) | 0.761 |
| | Female | 85 | 41 (24, 49) | 17.07 (13.21, 21.52) | 17.42 ± 5.88 | -0.60 ± 1.71 | **0.003** |
| **Fat(%)** | Total | 150 | 39 (23, 50) | 26.76 (21.72, 32.03) | 26.99 (20.91, 32.40) | -0.44 ± 2.30 | **0.017** |
| | Male | 65 | 34 (22, 53) | 23.12 (14.93, 27.61) | 22.23 (14.34, 27.92) | -0.13 ± 2.15 | 0.599 |
| | Female | 85 | 41 (24, 49) | 30.63 ± 6.86 | 29.94 ± 6.95 | -0.68 ± 2.39 | **0.010** |
| **Muscle mass(kg)** | Total | 150 | 39 (23, 50) | 40.40 (36.27, 51.17) | 40.17 (36.28, 51.17) | -0.11 (-1.05, 0.73) | 0.463 |
| | Male | 65 | 34 (22, 53) | 52.33 ± 5.27 | 52.22 ± 5.26 | -0.11 ± 1.21 | 0.476 |
| | Female | 85 | 41 (24, 49) | 36.73 ± 3.15 | 36.77 ± 3.04 | 0.04 ± 1.29 | 0.794 |
| **Muscle(%)** | Total | 150 | 39 (23, 50) | 68.54 (63.11, 73.46) | 67.82 (62.98, 74.53) | 0.58 ± 2.61 | **0.014** |

| | | | | | | | |
|---|---|---|---|---|---|---|---|
| | Male | 65 | 34 (22, 53) | 72.10 (68.21, 80.52) | 73.10 (67.23, 81.74) | 0.17 ± 2.59 | 0.741 |
| | Female | 85 | 41 (24, 49) | 64.38 ± 6.52 | 65.27 ± 6.82 | 0.89 ± 2.59 | **0.002** |
| **BMD(g/cm$^3$)** | Total | 150 | 39 (23, 50) | 1.15 ± 0.09 | 1.14 (1.10, 1.20) | -0.00 (-0.01, 0.01) | **0.041** |
| | Male | 65 | 34 (22, 53) | 1.20 ± 0.07 | 1.18 (1.14, 1.24) | -0.00 (-0.01, 0.01) | **0.033** |
| | Female | 85 | 41 (24, 49) | 1.12 (1.08, 1.16) | 1.11 (1.08, 1.14) | -0.00 (-0.01, 0.01) | 0.416 |
| **BMC(kg)** | Total | 150 | 39 (23, 50) | 2.63 ± 0.43 | 2.62 ± 0.42 | -0.00 (-0.04, 0.03) | 0.116 |
| | Male | 65 | 34 (22, 53) | 2.96 ± 0.34 | 2.96 ± 0.34 | -0.01 (-0.04, 0.03) | 0.346 |
| | Female | 85 | 41 (24, 49) | 2.37 ± 0.28 | 2.36 ± 0.28 | -0.01 ± 0.06 | 0.207 |
| **Cycling group** | | | | | | | |
| **Weight(kg)** | Total | 133 | 37 (21, 50) | 61.10 (55.30, 68.20) | 60.60 (54.80, 68.40) | -0.10 (-1.30, 1.10) | 0.566 |
| | Male | 52 | 26 (20.8, 43.3) | 70.13 ± 11.37 | 70.76 ± 11.38 | 0.75 (-0.33, 1.95) | 0.078 |
| | Female | 81 | 37 (21, 50) | 57.83 ± 7.15 | 57.20 ± 6.68 | -0.40 (-1.70, 0.40) | **0.002** |
| **Fat mass(kg)** | Total | 133 | 37 (21, 50) | 17.03 ± 7.17 | 16.80 ± 6.65 | -0.23 ± 2.06 | 0.196 |

| | | | | | | | |
|---|---|---|---|---|---|---|---|
| | Male | 52 | 26 (20.8, 43.3) | 12.44 (7.87, 20.54) | 14.79 ± 8.22 | 0.92 (-0.48, 1.84) | **0.023** |
| | Female | 81 | 37 (21, 50) | 18.81 ± 5.53 | 18.09 ± 5.07 | -0.73 ± 1.65 | **0.000** |
| **Fat(%)** | Total | 133 | 37 (21, 50) | 28.44 (19.97, 34.69) | 28.53 (20.54, 33.15) | -0.24 ± 2.52 | 0.405 |
| | Male | 52 | 26 (20.8, 43.3) | 19.27 ± 9.41 | 19.91 ± 8.88 | 0.65 ± 2.63 | 0.081 |
| | Female | 81 | 37 (21, 50) | 33.14 (27.43, 36.97) | 31.17 ± 6.17 | -0.82 ± 2.29 | **0.003** |
| **Muscle mass(kg)** | Total | 133 | 37 (21, 50) | 39.28 (35.64, 50.00) | 39.28 (35.58, 49.61) | -0.12 (-0.91, 0.80) | 0.698 |
| | Male | 52 | 26 (20.8, 43.3) | 52.67 ± 6.28 | 52.70 ± 6.33 | -0.35 (-1.11, 0.93) | 0.909 |
| | Female | 81 | 37 (21, 50) | 36.20 ± 3.36 | 36.27 ± 3.35 | 0.07 ± 1.28 | 0.646 |
| **Muscle(%)** | Total | 133 | 37 (21, 50) | 66.92 (60.27, 74.66) | 66.48 (61.61, 74.95) | 0.15 ± 2.63 | 0.795 |
| | Male | 52 | 26 (20.8, 43.3) | 76.11 ± 9.17 | 75.41 ± 8.65 | -1.13 (-2.49, 0.60) | **0.068** |
| | Female | 81 | 37 (21, 50) | 61.67 (58.17, 67.50) | 63.82 ± 5.85 | 0.70 ± 2.44 | **0.018** |
| **BMD(g/cm$^3$)** | Total | 133 | 37 (21, 50) | 1.14 ± 0.10 | 1.14 ± 0.10 | -0.00 ± 0.02 | 0.256 |
| | Male | 52 | 26 (20.8, 43.3) | 1.19 ± 0.10 | 1.19 ± 0.10 | 0.00 ± 0.02 | 0.692 |

| | | | | | | | |
|---|---|---|---|---|---|---|---|
| | Female | 81 | 37 (21, 50) | 1.11 ± 0.09 | 1.11 ± 0.09 | -0.00 ± 0.01 | 0.068 |
| **BMC(kg)** | Total | 133 | 37 (21, 50) | 2.58 ± 0.49 | 2.58 ± 0.49 | 0.00 (-0.04, 0.04) | 0.821 |
| | Male | 52 | 26 (20.8, 43.3) | 2.95 ± 0.45 | 2.96 ± 0.45 | 0.01 (-0.03, 0.05) | 0.260 |
| | Female | 81 | 37 (21, 50) | 2.34 ± 0.34 | 2.34 ± 0.34 | -0.00 (-0.04, 0.03) | 0.499 |
| **Combined group** | | | | | | | |
| **Weight(kg)** | Total | 49 | 28 (24, 36) | 60.30 (53.20, 71.70) | 60.40 (53.00, 71.10) | -0.13 ± 1.85 | 0.955 |
| | Male | 23 | 28.0 ± 6.2 | 71.06 ± 12.11 | 70.29 ± 11.32 | -0.10 (-2.30, 0.80) | 0.090 |
| | Female | 26 | 29 (23.3, 37) | 54.91 ± 7.13 | 55.35 ± 7.41 | 0.44 ± 1.42 | 0.129 |
| **Fat mass(kg)** | Total | 49 | 28 (24, 36) | 15.94 ± 7.06 | 15.62 ± 6.95 | -0.20 (-1.14, 0.43) | 0.175 |
| | Male | 23 | 28.0 ± 6.2 | 18.21 (6.57, 23.25) | 16.21 (6.86, 22.99) | -0.72 ± 2.00 | 0.142 |
| | Female | 26 | 29 (23.3, 37) | 16.18 ± 5.33 | 16.38 (11.23, 18.72) | 0.03 ± 1.15 | 0.764 |
| **Fat(%)** | Total | 49 | 28 (24, 36) | 27.10 (20.50, 31.10) | 24.63 ± 8.82 | -0.43 ± 1.73 | 0.085 |
| | Male | 23 | 28.0 ± 6.2 | 25.40 (10.15, 28.85) | 23.30 (10.65, 27.95) | -0.62 ± 2.05 | 0.153 |

| | | | | | | | |
|---|---|---|---|---|---|---|---|
| | Female | 26 | 29 (23.3, 37) | 28.92 ± 6.25 | 28.66 ± 6.74 | -0.26 ± 1.41 | 0.351 |
| **Muscle mass(kg)** | Total | 49 | 28 (24, 36) | 40.94 (36.15, 51.89) | 42.18 (36.27, 52.35) | 0.27 (-0.26, 1.13) | **0.011** |
| | Male | 23 | 28.0 ± 6.2 | 52.55 ± 4.66 | 52.84 ± 4.39 | 0.29 ± 1.43 | 0.343 |
| | Female | 26 | 29 (23.3, 37) | 36.39 ± 3.14 | 36.89 ± 3.16 | 0.27 (-0.10, 1.09) | **0.006** |
| **Muscle(%)** | Total | 49 | 28 (24, 36) | 68.54 (64.34, 75.21) | 71.57 ± 8.91 | 0.78 ± 1.95 | **0.011** |
| | Male | 23 | 28.0 ± 6.2 | 71.19 (67.28, 84.89) | 74.12 (68.43, 85.81) | 1.14 ± 2.30 | **0.021** |
| | Female | 26 | 29 (23.3, 37) | 66.81 ± 5.90 | 67.27 ± 6.49 | 0.46 ± 1.55 | 0.143 |
| **BMD(g/cm$^3$)** | Total | 49 | 28 (24, 36) | 1.17 ± 0.10 | 1.17 ± 0.09 | 0.00 (-0.01, 0.01) | 0.776 |
| | Male | 23 | 28.0 ± 6.2 | 1.22 ± 0.10 | 1.21 ± 0.10 | 0.00 (-0.01, 0.01) | 0.771 |
| | Female | 26 | 29 (23.3, 37) | 1.13 ± 0.07 | 1.13 ± 0.07 | -0.00 (-0.01, 0.01) | 0.360 |
| **BMC(kg)** | Total | 49 | 28 (24, 36) | 2.56 ± 0.45 | 2.57 ± 0.45 | 0.01 ± 0.05 | 0.189 |
| | Male | 23 | 28.0 ± 6.2 | 2.88 ± 0.38 | 2.89 ± 0.38 | 0.01 ± 0.07 | 0.532 |
| | Female | 26 | 29 (23.3, 37) | 2.28 ± 0.30 | 2.29 ± 0.30 | 0.01 ± 0.04 | 0.144 |

| | | | | | | | |
|---|---|---|---|---|---|---|---|
| **Control group** | | | | | | | |
| **Weight(kg)** | Total | 74 | 39 (26, 54.8) | 63.79 ± 12.30 | 64.73 ± 12.45 | 0.94 ± 1.80 | **0.000** |
| | Male | 29 | 32 (25, 59) | 71.56 ± 11.59 | 72.42 ± 12.10 | 0.87 ± 1.70 | **0.011** |
| | Female | 45 | 43 (30, 54) | 58.79 ± 10.01 | 59.78 ± 10.01 | 0.98 ± 1.87 | **0.001** |
| **Fat mass(kg)** | Total | 74 | 39 (26, 54.8) | 18.38 ± 7.41 | 19.10 ± 7.42 | 0.71 ± 1.40 | **0.000** |
| | Male | 29 | 32 (25, 59) | 15.98 ± 8.06 | 16.62 ± 8.13 | 0.64 ± 1.16 | **0.006** |
| | Female | 45 | 43 (30, 54) | 19.93 ± 6.60 | 20.69 ± 6.53 | 0.76 ± 1.54 | **0.002** |
| **Fat(%)** | Total | 74 | 39 (26, 54.8) | 30.00 (22.45, 35.25) | 31.30 (22.88, 35.42) | 0.60 (-0.40, 1.75) | **0.005** |
| | Male | 29 | 32 (25, 59) | 21.27 ± 8.20 | 21.84 ± 8.06 | 0.58 ± 1.48 | **0.044** |
| | Female | 45 | 43 (30, 54) | 32.98 ± 6.53 | 33.81 ± 6.18 | 0.83 ± 2.24 | **0.017** |
| **Muscle mass(kg)** | Total | 74 | 39 (26, 54.8) | 40.99 (34.79, 49.91) | 40.43 (35.20, 50.91) | 0.23 ± 1.15 | 0.068 |
| | Male | 29 | 32 (25, 59) | 52.49 ± 5.22 | 52.67 ± 5.26 | 0.18 ± 1.17 | 0.414 |
| | Female | 45 | 43 (30, 54) | 36.13 ± 3.88 | 36.40 ± 4.10 | 0.27 ± 1.15 | 0.124 |

| | | | | | | | |
|---|---|---|---|---|---|---|---|
| **Muscle(%)** | Total | 74 | 39 (26, 54.8) | 64.96 (60.28, 72.99) | 64.24 (60.32, 72.50) | -0.75 ± 2.10 | **0.009** |
| | Male | 29 | 32 (25, 59) | 74.37 ± 7.92 | 73.66 ± 7.76 | -0.71 ± 2.04 | 0.072 |
| | Female | 45 | 43 (30, 54) | 61.85 (57.09, 65.01) | 61.54 ± 5.60 | -0.77 ± 2.16 | **0.035** |
| **BMD(g/cm³)** | Total | 74 | 39 (26, 54.8) | 1.11 ± 0.08 | 1.11 ± 0.08 | 0.00 (-0.01, 0.01) | 0.164 |
| | Male | 29 | 32 (25, 59) | 1.14 ± 0.07 | 1.15 ± 0.08 | 0.00 ± 0.02 | 0.154 |
| | Female | 45 | 43 (30, 54) | 1.08 ± 0.07 | 1.09 ± 0.07 | 0.00 (-0.01, 0.01) | 0.511 |
| **BMC(kg)** | Total | 74 | 39 (26, 54.8) | 2.44 ± 0.39 | 2.45 ± 0.40 | 0.01 ± 0.07 | 0.275 |
| | Male | 29 | 32 (25, 59) | 2.78 ± 0.30 | 2.79 ± 0.30 | 0.01 ± 0.07 | 0.493 |
| | Female | 45 | 43 (30, 54) | 2.23 ± 0.28 | 2.24 ± 0.28 | 0.01 ± 0.07 | 0.404 |

Data are presented as mean ± SD or median (IQR), depending on the distribution. Comparisons between baseline and 16-week follow-up values for each exercise modality were conducted using paired t-tests (for normally distributed data) or Wilcoxon signed-rank tests (for non-normally distributed data).

Δ represents the difference between Week 16 and Baseline (Week 16 – Baseline).

**Bolded P values** indicate statistical significance ($P < 0.05$).

**Supplement Table S6. Associations of *FST* rs3797296/rs3797297 with the 16-Week Exercise-Induced Changes in Body Composition and Bone Mineral Parameters**

| | Sex (N) | rs3797296 | | | rs3797297 | | |
|---|---|---|---|---|---|---|---|
| | | **Beta (95% CI)** | ***P*** | ***Adj_P*** | **Beta (95% CI)** | ***P*** | ***Adj_P*** |
| **Δ_Fat(kg)** | Total (N=470) | -0.05 (-0.96, 0.85) | 0.906 | 0.967 | -0.21 (-1.12, 0.70) | 0.652 | 0.899 |
| | Male (N=208) | 0.46 (-1.13, 2.04) | 0.573 | 0.825 | -0.64 (-2.16, 0.89) | 0.414 | 0.753 |
| | Female (N=262) | -0.41 (-1.46, 0.65) | 0.453 | 0.998 | 0.16 (-0.97, 1.28) | 0.783 | 0.955 |
| **Δ_Fat(%)** | Total (N=470) | 0.00 (-1.12, 1.12) | 0.998 | 0.741 | -0.24 (-1.37, 0.89) | 0.677 | 0.917 |
| | Male (N=208) | 0.76 (-0.96, 2.49) | 0.385 | 0.819 | -0.88 (-2.56, 0.80) | 0.304 | 0.646 |
| | Female (N=262) | -0.43 (-1.89, 1.03) | 0.565 | 0.738 | 0.18 (-1.37, 1.73) | 0.819 | 0.955 |
| **Δ_Muscle(kg)** | Total (N=470) | -0.16 (-0.82, 0.49) | 0.625 | 0.879 | 0.12 (-0.54, 0.78) | 0.718 | 0.938 |
| | Male (N=208) | -0.58 (-1.70, 0.54) | 0.309 | 0.650 | 0.05 (-1.02, 1.13) | 0.924 | 0.973 |
| | Female (N=262) | 0.19 (-0.61, 0.99) | 0.644 | 0.896 | 0.08 (-0.77, 0.93) | 0.857 | 0.964 |

| | | | | | | | |
|---|---|---|---|---|---|---|---|
| **Δ_Muscle(%)** | Total (N=470) | -0.17 (-1.41, 1.07) | 0.791 | 0.955 | 0.02 (-1.24, 1.28) | 0.976 | 0.991 |
| | Male (N=208) | -1.32 (-3.40, 0.76) | 0.214 | 0.572 | 0.95 (-1.05, 2.96) | 0.351 | 0.694 |
| | Female (N=262) | 0.57 (-0.98, 2.11) | 0.473 | 0.759 | -0.70 (-2.34, 0.95) | 0.406 | 0.753 |
| **Δ_BMD(g/cm$^3$)** | Total (N=470) | 0.00 (-0.02, 0.02) | 0.974 | 0.991 | -0.01 (-0.03, 0.01) | 0.287 | 0.646 |
| | Male (N=208) | 0.00 (-0.03, 0.04) | 0.899 | 0.964 | -0.02 (-0.05, 0.02) | 0.268 | 0.636 |
| | Female (N=262) | 0.00 (-0.01, 0.01) | 0.781 | 0.955 | 0.00 (-0.01, 0.01) | 0.835 | 0.956 |
| **Δ_BMC(kg)** | Total (N=470) | 0.00 (-0.03, 0.04) | 0.807 | 0.955 | 0.03 (0.00, 0.06) | 0.094 | 0.410 |
| | Male (N=208) | 0.04 (-0.02, 0.09) | 0.175 | 0.537 | -0.01 (-0.06, 0.05) | 0.799 | 0.955 |
| | Female (N=262) | -0.01 (-0.05, 0.03) | 0.508 | 0.782 | 0.05 (0.00, 0.09) | **0.036** | 0.218 |

GLM analyses were used to assess whether FST rs3797296/rs3797297 modifies the effects of the 16-week exercise intervention on body composition and bone mineral parameters. The dependent variables were the 16-week changes (Δ) in each outcome parameter. The independent variables included the interaction terms between rs3797296/rs3797297 and group factors (exercise intervention vs. control group). Analyses were conducted for the overall sample, as well as stratified by sex. All models were adjusted for age, baseline BMI, and respective baseline values of the outcome parameters.

**Beta (95% CI):** Regression coefficient and 95% confidence interval.

Bolded p values (< 0.05) represent significant findings before multiple comparison correction.

***Adj_p*: ** Benjamini-Hochberg – adjusted p value (< 0.05 indicates statistical significance; denoted by ┼).

**Supplement Table S7. Interactions between *FST* rs3797296/rs3797297 and Different Exercise Modalities on 16-Week Changes in Body Composition and Bone Mineral Parameters**

| | Sex | N | rs3797296 | | | rs3797297 | | |
|---|---|---|---|---|---|---|---|---|
| | | | Beta (95% CI) | *P* | *Adj_P* | Beta (95% CI) | *P* | *Adj_P* |
| **Hill group** | | | | | | | | |
| **Δ_Fat(kg)** | Total | 64 | -0.30 (-1.55, 0.95) | 0.638 | 0.767 | 0.34 (-0.92, 1.60) | 0.592 | 0.700 |
| | Male | 39 | 0.47 (-1.49, 2.43) | 0.636 | 0.827 | 0.12 (-1.75, 1.99) | 0.899 | 0.928 |
| | Female | 25 | -0.82 (-2.45, 0.82) | 0.326 | 0.496 | 0.49 (-1.33, 2.30) | 0.598 | 0.707 |
| **Δ_Fat(%)** | Total | 64 | 0.07 (-1.63, 1.48) | 0.927 | 0.994 | 0.61 (-0.95, 2.17) | 0.443 | 0.576 |
| | Male | 39 | 0.90 (-1.24, 3.05) | 0.409 | 0.556 | 0.18 (-1.87, 2.24) | 0.862 | 0.896 |
| | Female | 25 | -0.54 (-2.81, 1.74) | 0.645 | 0.762 | 0.77 (-1.75, 3.30) | 0.548 | 0.712 |
| **Δ_Muscle(kg)** | Total | 64 | -0.37 (-1.27, 0.53) | 0.421 | 0.736 | -0.54 (-1.45, 0.37) | 0.243 | 0.632 |
| | Male | 39 | -0.26 (-1.65, 1.14) | 0.719 | 0.965 | -0.91 (-2.24, 0.42) | 0.178 | 0.889 |
| | Female | 25 | -0.79 (-2.02, 0.43) | 0.205 | 0.444 | 0.18 (-1.19, 1.56) | 0.793 | 0.937 |
| **Δ_Muscle(%)** | Total | 64 | -0.16 (-1.88, 1.55) | 0.852 | 0.866 | -0.52 (-2.24, 1.21) | 0.557 | 0.725 |
| | Male | 39 | -1.10 (-3.65, 1.45) | 0.398 | 0.583 | 0.95 (-2.30, 2.60) | 0.904 | 0.904 |
| | Female | 25 | 0.35 (-2.06, 2.76) | 0.777 | 0.918 | -1.03 (-3.69, 1.64) | 0.451 | 0.651 |
| **Δ_BMD** | Total | 64 | -0.01 (-0.03, 0.01) | 0.367 | 0.911 | 0.00 (-0.03, 0.02) | 0.703 | 0.944 |
| **(g/cm3)** | Male | 39 | -0.01 (-0.05, 0.04) | 0.695 | 0.905 | -0.01 (-0.06, 0.03) | 0.573 | 0.982 |
| | Female | 25 | -0.01 (-0.03, 0.01) | 0.159 | 0.680 | 0.00 (-0.02, 0.02) | 0.925 | 0.925 |

| | | | | | | | | |
|---|---|---|---|---|---|---|---|---|
| **Δ_BMC(kg)** | Total | 64 | 0.02 (-0.03, 0.06) | 0.434 | 0.994 | 0.04 (-0.01, 0.08) | 0.103 | 0.334 |
| | Male | 39 | 0.05 (-0.02, 0.11) | 0.195 | 0.422 | 0.00 (-0.07, 0.06) | 0.912 | 0.988 |
| | Female | 25 | 0.00 (-0.06, 0.07) | 0.936 | 0.936 | 0.07 (0.00, 0.13) | 0.066 | 0.115 |
| **Running group** | | | | | | | | |
| **Δ_Fat(kg)** | Total | 150 | 0.23 (-0.78, 1.25) | 0.649 | 0.767 | -0.24 (-1.32, 0.84) | 0.666 | 0.721 |
| | Male | 65 | 0.75 (-1.00, 2.50) | 0.402 | 0.653 | -0.59 (-2.29, 1.11) | 0.495 | 0.715 |
| | Female | 85 | -0.14 (-1.33, 1.04) | 0.814 | 0.814 | -0.04 (-1.43, 1.36) | 0.960 | 0.960 |
| **Δ_Fat(%)** | Total | 150 | 0.27 (-0.99, 1.52) | 0.677 | 0.989 | -0.40 (-1.74, 0.94) | 0.559 | 0.661 |
| | Male | 65 | 0.93 (-1.00, 2.85) | 0.345 | 0.556 | -0.91 (-2.78, 0.96) | 0.340 | 0.553 |
| | Female | 85 | -0.16 (-1.81, 1.48) | 0.845 | 0.916 | -0.18 (-2.12, 1.76) | 0.857 | 0.929 |
| **Δ_Muscle(kg)** | Total | 150 | -0.13 (-0.85, 0.60) | 0.733 | 0.858 | 0.24 (-0.54, 1.02) | 0.541 | 0.703 |
| | Male | 65 | -0.68 (-1.94, 0.58) | 0.290 | 0.750 | 0.31 (-0.91, 1.53) | 0.615 | 0.889 |
| | Female | 85 | 0.33 (-0.56, 1.22) | 0.467 | 0.594 | 0.00 (-1.06, 1.05) | 0.996 | 0.996 |
| **Δ_Muscle(%)** | Total | 150 | -1.97 (-4.26, 0.33) | 0.558 | 0.852 | 0.10 (-1.38, 1.58) | 0.894 | 0.918 |
| | Male | 65 | 0.55 (-1.20, 2.29) | 0.093 | 0.241 | 1.12 (-1.11, 3.35) | 0.324 | 0.664 |
| | Female | 85 | 0.57 (-0.98, 2.11) | 0.540 | 0.701 | -0.68 (-2.72, 1.37) | 0.518 | 0.673 |
| **Δ_BMD** | Total | 150 | 0.01 (-0.01, 0.02) | 0.593 | 0.911 | -0.02 (-0.04, 0.00) | **0.044** | 0.281 |
| **(g/cm3)** | Male | 65 | 0.01 (-0.03, 0.05) | 0.683 | 0.905 | -0.04 (-0.08, 0.00) | **0.044** | 0.365 |
| | Female | 85 | 0.00 (-0.01, 0.01) | 0.883 | 0.956 | 0.00 (-0.01, 0.02) | 0.871 | 0.925 |
| **Δ_BMC(kg)** | Total | 150 | 0.00 (-0.04, 0.04) | 0.974 | 0.994 | 0.03 (-0.01, 0.07) | 0.183 | 0.339 |
| | Male | 65 | 0.01 (-0.05, 0.07) | 0.693 | 0.842 | -0.02 (-0.08, 0.04) | 0.488 | 0.988 |

| | | | | | | | | |
|---|---|---|---|---|---|---|---|---|
| | Female | 85 | -0.01 (-0.05, 0.04) | 0.739 | 0.936 | 0.06 (0.01, 0.11) | **0.032** | 0.083 |
| **Cycling group** | | | | | | | | |
| **Δ_Fat(kg)** | Total | 133 | -0.34 (1.37, 0.69) | 0.517 | 0.747 | -0.88 (-1.96, 0.20) | 0.111 | 0.241 |
| | Male | 52 | -0.05 (-1.86, 1.76) | 0.960 | 0.975 | -1.81 (-3.62, -0.01) | 0.049 | 0.211 |
| | Female | 81 | -0.53 (-1.73, 0.67) | 0.382 | 0.496 | -0.13 (-1.43, 1.17) | 0.845 | 0.915 |
| **Δ_Fat(%)** | Total | 133 | -0.26 (-1.54, 1.01) | 0.685 | 0.989 | -0.95 (-2.28, 0.39) | 0.165 | 0.268 |
| | Male | 52 | 0.36 (-1.61, 2.34) | 0.719 | 0.734 | -2.16 (-4.14, -0.18) | **0.033** | 0.142 |
| | Female | 81 | -0.60 (-2.27, 1.07) | 0.481 | 0.665 | -0.07 (-1.87, 1.73) | 0.94 | 0.94 |
| **Δ_Muscle(kg)** | Total | 133 | -0.29 (-1.03, 0.45) | 0.446 | 0.736 | 0.37 (-0.41, 1.15) | 0.352 | 0.689 |
| | Male | 52 | -1.00 (-2.29, 0.29) | 0.130 | 0.750 | 0.49 (-0.80, 1.78) | 0.455 | 0.889 |
| | Female | 81 | 0.18 (-0.72, 1.07) | 0.699 | 0.699 | 0.17 (-0.81, 1.15) | 0.734 | 0.937 |
| **Δ_Muscle(%)** | Total | 133 | 0.19 (-1.22, 1.59) | 0.797 | 0.866 | 0.66 (-0.82, 2.14) | 0.381 | 0.619 |
| | Male | 52 | -0.48 (-2.83, 1.87) | 0.691 | 0.691 | 1.90 (-0.46, 4.26) | 0.115 | 0.375 |
| | Female | 81 | 0.58 (-1.18, 2.34) | 0.519 | 0.701 | -0.19 (-2.08, 1.71) | 0.848 | 0.919 |
| **Δ_BMD** | Total | 133 | 0.00 (-0.02, 0.02) | 0.719 | 0.911 | 0.00 (-0.02, 0.02) | 0.647 | 0.944 |
| **(g/cm3)** | Male | 52 | 0.00 (-0.04, 0.05) | 0.822 | 0.905 | -0.01 (-0.05, 0.03) | 0.587 | 0.982 |
| | Female | 81 | -0.01 (-0.02, 0.01) | 0.383 | 0.680 | 0.00 (-0.02, 0.01) | 0.834 | 0.925 |
| **Δ_BMC(kg)** | Total | 133 | 0.00 (-0.04, 0.04) | 0.947 | 0.994 | 0.02 (-0.02, 0.06) | 0.258 | 0.350 |
| | Male | 52 | 0.06 (0.00, 0.12) | 0.060 | 0.324 | 0.01 (-0.05, 0.07) | 0.750 | 0.988 |
| | Female | 81 | -0.03 (-0.08, 0.02) | 0.198 | 0.809 | 0.03 (-0.02, 0.08) | 0.264 | 0.343 |
| **Combined group** | | | | | | | | |

| | | | | | | | | |
|---|---|---|---|---|---|---|---|---|
| **Δ_Fat(kg)** | Total | 49 | 0.09(-1.20, 1.39) | 0.887 | 0.960 | 0.83 (-0.58, 2.23) | 0.248 | 0.358 |
| | Male | 23 | 0.25 (-2.05, 2.55) | 0.830 | 0.975 | 0.45 (-1.65, 2.56) | 0.674 | 0.797 |
| | Female | 26 | -0.64 (-2.19, 0.91) | 0.419 | 0.496 | 1.58 (-0.36, 3.51) | 0.110 | 0.239 |
| **Δ_Fat(%)** | Total | 49 | 0.01 (-1.60, 1.62) | 0.994 | 0.994 | 0.79 (-0.95, 2.53) | 0.376 | 0.543 |
| | Male | 23 | 0.44 (-2.09, 2.97) | 0.734 | 0.734 | 0.15 (-2.16, 2.47) | 0.896 | 0.896 |
| | Female | 26 | -0.72 (-2.87, 1.43) | 0.512 | 0.665 | 1.55 (-1.16, 4.25) | 0.262 | 0.536 |
| **Δ_Muscle(kg)** | Total | 49 | 0.32 (-0.61, 1.25) | 0.504 | 0.736 | -0.02 (-1.03, 0.99) | 0.967 | 0.967 |
| | Male | 23 | 0.23 (-1.41, 1.87) | 0.785 | 0.965 | -0.02 (-1.52, 1.49) | 0.982 | 0.982 |
| | Female | 26 | 0.56 (-0.60, 1.72) | 0.345 | 0.594 | -0.26 (-1.73, 1.22) | 0.734 | 0.937 |
| **Δ_Muscle(%)** | Total | 49 | -0.21 (-1.99, 1.56) | 0.814 | 0.866 | -1.25 (-3.18, 0.68) | 0.204 | 0.378 |
| | Male | 23 | -0.88 (-3.89, 2.13) | 0.566 | 0.669 | -0.43 (-3.20, 2.33) | 0.758 | 0.821 |
| | Female | 26 | 0.83 (-1.45, 3.11) | 0.476 | 0.701 | -2.39 (-5.24, 0.47) | 0.101 | 0.263 |
| **Δ_BMD** | Total | 49 | 0.00 (-0.02, 0.03) | 0.694 | 0.911 | 0.00 (-0.03, 0.02) | 0.888 | 0.962 |
| **(g/cm3)** | Male | 23 | -0.01 (-0.06, 0.05) | 0.823 | 0.905 | 0.00 (-0.05, 0.05) | 0.974 | 0.982 |
| | Female | 26 | 0.01 (-0.01, 0.03) | 0.293 | 0.680 | -0.01 (-0.03, 0.01) | 0.383 | 0.731 |
| **Δ_BMC(kg)** | Total | 49 | 0.00 (-0.04, 0.05) | 0.848 | 0.994 | 0.04 (-0.01, 0.09) | 0.165 | 0.339 |
| | Male | 23 | 0.03 (-0.05, 0.11) | 0.484 | 0.699 | -0.01 (-0.08, 0.07) | 0.819 | 0.988 |
| | Female | 26 | -0.01 (-0.07, 0.05) | 0.776 | 0.936 | 0.07 (0.00, 0.14) | 0.059 | 0.115 |

GLM analyses were used to assess the interactions between FST rs3797296/rs3797297 and different exercise modalities on the 16-week changes in body composition and bone mineral parameters. The dependent variables were the 16-week changes in each outcome , and the independent variables were the interaction

terms between FST SNPs and specific exercise modalities. Analyses were conducted for the overall sample, as well as stratified by sex. All models were adjusted for age, baseline BMI, and baseline values of the respective outcome parameters.

**Beta (95% CI):** Regression coefficient and 95% confidence interval.

Bolded p values (< 0.05) represent significant findings before multiple comparison correction.

***Adj_p***: Benjamini-Hochberg ‐ adjusted p value (< 0.05 indicates statistical significance; denoted by ┼).

**Supplement Table S8. Baseline Serum Follistatin Levels by Sex and Group**

| Group | Sex | N | Follistatin | *P* |
|---|---|---|---|---|
| **Overall** | Male | 114 | 290.76 (199.91, 491.03) | **0.014** |
| | Female | 120 | 260.21 (170.91, 347.73) | |
| **Training group** | Male | 106 | 384.92 (253.38, 604.74) | **0.002** |
| | Female | 106 | 283.43 (208.95, 480.50) | |
| **Control group** | Male | 8 | 290.76 (199.91, 491.03) | 0.433 |
| | Female | 14 | 260.21 (170.91, 347.73) | |

Data are presented as mean ± SD or median (IQR), depending on the distribution. Comparisons of baseline serum follistatin levels by sex were performed using paired t-tests (for normally distributed data) or Wilcoxon signed-rank tests (for non-normally distributed data).

**Training group** refers to the group that underwent the 16-week exercise intervention, while the **Control group** refers to the non-intervention group.

**Bolded P values** indicate statistical significance ($P < 0.05$).

**Supplement Table S9. Changes in Serum Follistatin Following 16-Week Exercise Intervention by Exercise Modality and Sex**

| | Sex | N | Ages | Baseline | Week16 | Δ | *P* |
|---|---|---|---|---|---|---|---|
| **Training group** | | | | | | | |
| **Follistatin** | Total | 212 | 39.5 (23, 51) | 278.76 (184.00, 421.29) | 335.63 (222.12, 545.61) | 60.80 (-97.21, 169.87) | **0.004** |
| | Male | 106 | 34.5 (22, 49) | 304.63 (199.91, 528.89) | 384.92 (253.38, 604.74) | 57.66 (-84.04, 171.21) | **0.012** |
| | Female | 106 | 41 (29.3, 52) | 263.13 (173.48, 346.00) | 283.43 (208.95, 480.50) | 61.99 (-104.73, 158.37) | 0.113 |
| **Control group** | | | | | | | |
| **Follistatin** | Total | 22 | 43 (26, 56.8) | 255.71 (161.03, 314.16) | 213.96 (151.34, 413.81) | 44.90 (-104.58, 170.51) | 0.406 |
| | Male | 8 | 42.5 (20.8, 60) | 256.97 (214.40, 283.77) | 207.63 (125.22, 370.02) | -63.33 (-111.94, 125.82) | 0.977 |
| | Female | 14 | 43 (30.8, 54.5) | 249.97 (121.42, 352.04) | 213.96 (181.59, 413.81) | 83.39 (-78.10, 194.47) | 0.268 |
| **Hill group** | | | | | | | |
| **Follistatin** | Total | 50 | 46 (33, 53.5) | 252.11 (179.43, 314.13) | 297.70 (222.50, 380.31) | 56.87 (-15.68, 132.18) | **0.010** |
| | Male | 31 | 45 (29, 48.5) | 253.11 (195.11, 315.34) | 327.41 (216.50, 382.76) | 49.79 (-2.28, 131.31) | **0.021** |

| | | | | | | | |
|---|---|---|---|---|---|---|---|
| | Female | 19 | 50 (39.5, 55.5) | 240.35 ± 106.77 | 300.57 ± 158.57 | 70.08 (-74.89, 135.05) | 0.128 |
| **Running group** | | | | | | | |
| **Follistatin** | Total | 76 | 44 (23, 52) | 304.96 (178.84, 502.52) | 365.89 (253.04, 647.92) | 58.95 (-85.11, 193.85) | **0.043** |
| | Male | 37 | 44 (21, 55) | 475.44 (210.22, 839.37) | 572.72 (326.20, 852.51) | 62.12 (-78.35, 188.34) | 0.182 |
| | Female | 39 | 44 (32.5, 51) | 271.56 ± 121.67 | 275.74 (210.90, 526.11) | 55.79 (-89.04, 201.96) | 0.131 |
| **Cycling group** | | | | | | | |
| **Follistatin** | Total | 64 | 37 (20.8, 50.3) | 332.70 (205.49, 540.56) | 415.46 (205.39, 561.07) | 9.81 (-146.65, 175.96) | 0.867 |
| | Male | 26 | 21 (20, 37.8) | 418.46 (249.58, 732.75) | 478.49 (371.80, 597.82) | 75.81 (-117.32, 167.08) | 0.600 |
| | Female | 38 | 41 (24, 51.8) | 270.02 (179.97, 476.33) | 285.02 (161.51, 508.79) | -74.01 (-149.42, 183.52) | 0.841 |
| **Combined group** | | | | | | | |
| Follistatin | Total | 22 | 30 (23.3, 35.8) | 228.17 ± 96.29 | 304.99 ± 160.88 | 76.82 ± 193.22 | 0.076 |
| | Male | 12 | 33 (26.8, 35.5) | 195.06 ± 94.72 | 278.85 ± 199.95 | 83.80 ± 246.75 | 0.264 |
| | Female | 10 | 23.5 (22.3, 34.8) | 267.91 ± 86.32 | 336.36 ± 97.59 | 68.46 ± 112.06 | 0.085 |

Data are presented as mean ± SD or median (IQR), depending on the distribution. Comparisons between baseline and 16-week follow-up values were conducted

using paired t-tests (for normally distributed data) or Wilcoxon signed-rank tests (for non-normally distributed data).

Δ represents the difference between Week 16 and Baseline (Week 16 – Baseline).

**Training group** refers to the group that underwent the 16-week exercise intervention, while the **Control group** refers to the non-intervention group.

Data are presented broken down by Training group and exercise modality (Hill, Running, Cycling, Combined.), and by sex.

**Bolded P values** indicate statistical significance ($P < 0.05$).

**Supplement Table S10. Mediation Analysis of Follistatin Changes in the Relationship Between FST rs3797296/rs3797297 and Exercise Response**

| | Indirect effect （Δ_ Follistatin） | Sex | N | rs3797296 | | | rs3797297 | | |
|---|---|---|---|---|---|---|---|---|---|
| | | | | Coefficient | SE | P-Value | Coefficient | SE | P-Value |
| **Training group** | **Δ_Fat(kg)** | Male | 106 | 0.0001 | 0.075 | 0.999 | 0.001 | 0.107 | 0.994 |
| | | Female | 106 | 0.004 | 0.067 | 0.952 | 0.004 | 0.105 | 0.968 |
| | **Δ_Fat(%)** | Male | 106 | 0.0001 | 0.079 | 0.999 | -0.023 | 0.117 | 0.846 |
| | | Female | 106 | 0.012 | 0.098 | 0.901 | -0.032 | 0.162 | 0.843 |
| | **Δ_Muscle(kg)** | Male | 106 | 0.001 | 0.072 | 0.987 | 0.047 | 0.092 | 0.615 |
| | | Female | 106 | -0.008 | 0.061 | 0.895 | 0.039 | 0.087 | 0.654 |
| | **Δ_Muscle(%)** | Male | 106 | -0.002 | 0.110 | 0.989 | -0.005 | 0.164 | 0.976 |
| | | Female | 106 | -0.012 | 0.114 | 0.916 | 0.038 | 0.175 | 0.830 |
| | **Δ_BMD(g/cm$^3$)** | Male | 106 | 0.0002 | 0.002 | 0.935 | 0.003 | 0.003 | 0.336 |

| | | | | | | | | |
|---|---|---|---|---|---|---|---|---|
| | Female | 106 | -0.0002 | 0.001 | 0.867 | 0.002 | 0.002 | 0.370 |
| **Δ_BMC(g)** | Male | 106 | 0.295 | 4.269 | 0.945 | 2.117 | 5.403 | 0.695 |
| | Female | 106 | 0.220 | 2.540 | 0.931 | 0.051 | 4.039 | 0.990 |

The table shows whether SNPs (rs3797296 and rs3797297) influence changes in body composition and bone mineral parameters through changes in follistatin (Δ_ Follistatin), and whether this effect is modified by the training group.

**Training group** refers to the participants who underwent the 16-week exercise intervention.

**Supplement Table S11. Mediation Analysis of Follistatin Changes in the Relationship Between FST rs3797296/rs3797297 and Exercise Response by Exercise Modalities**

| | Indirect effect （Δ_ Follistatin） | Sex | N | rs3797296 | | | rs3797297 | | |
|---|---|---|---|---|---|---|---|---|---|
| | | | | Coefficient | SE | P-Value | Coefficient | SE | P-Value |
| **Hill group** | **Δ_Fat(kg)** | Male | 31 | 0.028 | 0.123 | 0.822 | 0.027 | 0.167 | 0.870 |
| | | Female | 19 | -0.087 | 0.318 | 0.784 | 0.124 | 0.567 | 0.826 |

| | | | | | | | | | |
|---|---|---|---|---|---|---|---|---|---|
| | **Δ_Fat(%)** | Male | 31 | 0.030 | 0.120 | 0.800 | 0.037 | 0.157 | 0.815 |
| | | Female | 19 | -0.281 | 0.551 | 0.610 | 0.207 | 0.552 | 0.708 |
| | **Δ_Muscle(kg)** | Male | 31 | 0.019 | 0.090 | 0.832 | -0.066 | 0.137 | 0.632 |
| | | Female | 19 | 0.089 | 0.256 | 0.727 | 0.244 | 0.416 | 0.557 |
| | **Δ_Muscle(%)** | Male | 31 | 0.001 | 0.138 | 0.994 | -0.044 | 0.207 | 0.831 |
| | | Female | 19 | 0.251 | 0.621 | 0.686 | -0.100 | 0.726 | 0.891 |
| | **Δ_BMD(g/cm$^3$)** | Male | 31 | 0.0004 | 0.002 | 0.846 | -0.0004 | 0.002 | 0.862 |
| | | Female | 19 | -0.001 | 0.005 | 0.902 | -0.001 | 0.006 | 0.915 |
| | **Δ_BMC(g)** | Male | 31 | 1.228 | 5.683 | 0.829 | -0.839 | 7.558 | 0.912 |
| | | Female | 19 | -8.063 | 16.256 | 0.620 | 2.118 | 23.748 | 0.929 |
| **Running group** | **Δ_Fat(kg)** | Male | 37 | -0.102 | 0.194 | 0.601 | 0.242 | 0.298 | 0.417 |
| | | Female | 39 | -0.044 | 0.131 | 0.737 | 0.037 | 0.142 | 0.793 |
| | **Δ_Fat(%)** | Male | 37 | -0.091 | 0.203 | 0.654 | 0.252 | 0.305 | 0.409 |

| | | | | | | | | |
|---|---|---|---|---|---|---|---|---|
| | | Female | 39 | -0.056 | 0.209 | 0.790 | 0.100 | 0.261 | 0.702 |
| | **Δ_Muscle(kg)** | Male | 37 | -0.003 | 0.097 | 0.978 | -0.023 | 0.163 | 0.886 |
| | | Female | 39 | -0.006 | 0.097 | 0.951 | -0.058 | 0.127 | 0.650 |
| | **Δ_Muscle(%)** | Male | 37 | 0.062 | 0.231 | 0.789 | -0.170 | 0.388 | 0.660 |
| | | Female | 39 | 0.049 | 0.218 | 0.824 | -0.135 | 0.292 | 0.644 |
| | **Δ_BMD($g/cm^3$)** | Male | 37 | 0.001 | 0.001 | 0.588 | -0.002 | 0.002 | 0.398 |
| | | Female | 39 | 0.000 | 0.003 | 0.956 | -0.002 | 0.004 | 0.605 |
| | **Δ_BMC(g)** | Male | 37 | -2.137 | 5.039 | 0.671 | 5.232 | 7.912 | 0.508 |
| | | Female | 39 | -1.224 | 4.121 | 0.766 | 0.431 | 5.444 | 0.937 |
| **Cycling group** | **Δ_Fat(kg)** | Male | 26 | -0.008 | 0.175 | 0.963 | -0.057 | 0.210 | 0.786 |
| | | Female | 38 | 0.088 | 0.163 | 0.590 | 0.107 | 0.205 | 0.601 |
| | **Δ_Fat(%)** | Male | 26 | -0.0005 | 0.186 | 0.998 | -0.053 | 0.227 | 0.815 |
| | | Female | 38 | 0.107 | 0.232 | 0.644 | 0.150 | 0.283 | 0.596 |

| | | | | | | | | | |
|---|---|---|---|---|---|---|---|---|---|
| | **Δ_Muscle(kg)** | Male | 26 | 0.022 | 0.159 | 0.891 | -0.028 | 0.203 | 0.888 |
| | | Female | 38 | -0.001 | 0.110 | 0.991 | -0.053 | 0.131 | 0.683 |
| | **Δ_Muscle(%)** | Male | 26 | -0.001 | 0.271 | 0.997 | 0.031 | 0.305 | 0.919 |
| | | Female | 38 | -0.169 | 0.266 | 0.525 | -0.178 | 0.317 | 0.575 |
| | **Δ_BMD(g/cm$^3$)** | Male | 26 | -0.0005 | 0.002 | 0.777 | -0.0003 | 0.002 | 0.877 |
| | | Female | 38 | -0.001 | 0.002 | 0.553 | -0.001 | 0.002 | 0.586 |
| | **Δ_BMC(g)** | Male | 26 | -2.176 | 10.750 | 0.840 | -1.306 | 10.019 | 0.896 |
| | | Female | 38 | -0.521 | 5.285 | 0.921 | 0.775 | 4.802 | 0.872 |
| **Combined group** | **Δ_Fat(kg)** | Male | 12 | 0.217 | 8.841 | 0.980 | -0.029 | 10.460 | 0.998 |
| | | Female | 10 | -3.768 | 68.196 | 0.956 | 3.245 | 34.591 | 0.925 |
| | **Δ_Fat(%)** | Male | 12 | 0.525 | 9.896 | 0.958 | -0.267 | 11.192 | 0.981 |
| | | Female | 10 | 1.919 | 44.528 | 0.966 | -0.962 | 36.378 | 0.979 |
| | **Δ_Muscle(kg)** | Male | 12 | -0.001 | 9.508 | 1.000 | -0.023 | 12.633 | 0.999 |

| | | | | | | | | |
|---|---|---|---|---|---|---|---|---|
| | Female | 10 | -0.243 | 4.550 | 0.957 | 0.108 | 9.170 | 0.991 |
| **Δ_Muscle(%)** | Male | 12 | 0.405 | 10.900 | 0.970 | 0.232 | 9.975 | 0.981 |
| | Female | 10 | -1.794 | 55.736 | 0.974 | -0.611 | 17.478 | 0.972 |
| **Δ_BMD(g/cm$^3$)** | Male | 12 | 0.008 | 0.243 | 0.972 | 0.014 | 0.445 | 0.976 |
| | Female | 10 | 0.015 | 0.304 | 0.961 | -0.024 | 0.272 | 0.929 |
| **Δ_BMC(g)** | Male | 12 | -3.974 | 98.110 | 0.968 | -11.180 | 237.260 | 0.962 |
| | Female | 10 | -26.194 | 1254.570 | 0.983 | 7.973 | 1562.075 | 0.996 |

The table shows whether SNPs (rs3797296 and rs3797297) influence changes in body composition and bone mineral parameters through changes in follistatin (Δ_Follistatin) across different exercise modalities.
